# Integrated Microfluidic CRISPR-Based Diagnostic Device for Rapid Testing of HPVs

**DOI:** 10.3390/bios16100571

**Published:** 2026-10-11

**Authors:** Tianci Wang, Lingyun Li, Yunxiang Wang, Yongjun Han, Teng Li, Chaoran Yu, Yufeng Lou, Zhen Rong, Kai Lin, Shengqi Wang

**Affiliations:** 1 Bioinformatics Center of AMMS, Beijing 100850, China; 2 The Second Affiliated Hospital of Henan University of Traditional Chinese Medicine, Zhengzhou 450003, China; 3 Department of Pathology, Air Force Medical Center, Air Force Medical University, Beijing 100142, China; 4 Department of Laboratory Medicine, The First Affiliated Hospital, Zhejiang University School of Medicine, Hangzhou 310003, China; 5 Department of Clinical Laboratory, Air Force Medical Center, Air Force Medical University, Beijing 100142, China; 6 State Key Laboratory of Kidney Diseases, Beijing 100853, China

**Keywords:** human papillomavirus, microfluidic, CRISPR-based diagnostic, detection device

## Abstract

Screening for high-risk human papillomavirus (HR-HPV) is a pivotal strategy for the prevention of invasive cervical cancer, particularly in resource-constrained settings. In this study, we developed a CRISPR-based compact automated real-time detection system, termed Ccard, for rapid testing of HPV16, HPV18, and the internal reference gene GAPDH through a streamlined workflow. A simple room-temperature treatment efficiently releases nucleic acids directly from swab samples, bypassing the need for extraction. The mixture was driven through the channels and loaded into the reaction chambers on the microfluidic chip by a vacuum pump. The lyophilized reagents in the reaction zone of the microfluidic chip were redissolved. The system subsequently performs a single-step RPA-CRISPR/Cas13a reaction, with real-time detection enabled by an Internet of Things device. The assay achieved detection within 30 min, with a limit of detection (LOD) of 5 copies/µL for both HPV16 and HPV18. Validation with clinical samples demonstrated high sensitivity (96.8% for HPV16 and 100% for HPV18) and 100% specificity. These results highlight the reliability, precision, and strong potential of the Ccard system for rapid testing of HR-HPVs.

## 1. Introduction

Cervical cancer poses a significant public health challenge, as it is the fourth most common cancer among women worldwide [1]. Research has demonstrated that approximately 70% of cases are associated with persistent infections caused by high-risk HPV (human papillomavirus) types 16 and 18 [2,3]. HPV16 is the most prevalent oncogenic type, accounting for nearly half of all cases worldwide, while HPV18 accounts for approximately 16.5% [4,5]. Early diagnosis and vaccination have been demonstrated to be effective in the prevention of HPV infections and the reduction in cervical cancer risk [3]. However, traditional methods, such as PCR, are characterized by their high sensitivity, requiring the use of specialized laboratories, advanced equipment, and skilled staff [6,7,8,9]. This limitation restricts their application in point-of-care testing, primary healthcare, and underserved regions [10]. According to the data from the International Agency for Research on Cancer (IARC), cervical cancer has a greater impact on developing countries, with incidence and mortality rates being 1.7 and 2.4 times higher [11], respectively, than in developed nations. This disparity is primarily attributed to the limited access to screening programs in developing countries. The ASSURED (Affordable, Sensitive, Specific, User-friendly, Rapid, Equipment-free, Deliverable) criteria are a set of characteristics that define an ideal point-of-care testing technology [12,13]. Developing POCT technologies that meet these ASSURED criteria has become a global priority in the effort to improve cervical cancer prevention and management [14,15,16,17].

Clustered regularly interspaced short palindromic repeats/Cas-based diagnostics (CRISPR-Dx) has emerged as a critical tool in molecular detection owing to its high sensitivity and specificity [18,19]; however, its practical applications frequently necessitate reliance on pre-amplification steps (e.g., Recombinase Polymerase Amplification (RPA)) to enhance target detection sensitivity. The conventional two-step method requires physical separation of the amplification and CRISPR detection processes, which not only elevates the risk of aerosol contamination leading to false-positive results during operation but also impedes rapid quantitative analysis. To address this challenge, one-pot assay strategies have gained significant research focus: for instance, the nested RPA and CRISPR/Cas12a reactions are performed sequentially in separate wells on a disc-shaped microfluidic chip by controlling different rotational speeds [20]; temperature gradient regulation (TRACER) coordinates the reaction activity of RPA and CRISPR/Cas12b [21]; or droplet-based microfluidic pairing (DIMERIC) achieves spatiotemporal separation of amplification and detection [22,23]. Nonetheless, these approaches rely on complex microfluidic chips or precision control instruments. We have recently established a highly sensitive single-step RPA-CRISPR/Cas13a detection system [24], which is expected to simplify the detection process and microfluidic chip design, shorten reaction time, and enhance detection performance.

CRISPR-based diagnostics show considerable potential for point-of-care testing; nevertheless, the non-specific trans-cleavage of Cas enzymes presents a challenge to their effectiveness in multiplex target analysis. To address this challenge, two primary strategies have been developed. The initial approach utilizes orthogonal enzyme systems for the purpose of target discrimination. A notable example is SHERLOCK.V2, which employs the distinct recognition specificities of LwaCas13a, PsmCas13b, CcaCas13b, and AsCas12a to construct an orthogonal diagnostic system. This system facilitates the concurrent detection of up to four targets, thereby enhancing multiplexing capabilities while minimizing cross-reactivity [25]. The second approach utilizes microfluidic chip technology to achieve multiplex detection through physical compartmentalization. This method offers even greater advantages in miniaturization, integration, and automation, making it particularly better suited for multi-channel detection. The employment of centrifugal or channel-based chip designs facilitates the effective isolation of reaction units, thereby reducing nonspecific interference and enhancing detection accuracy. Nevertheless, some microfluidic solutions rely on precision motor drives, which can make the system more complex [26]. Alternatively, the lower liquid dispensing efficiency of microfluidic chips further restricts their applicability in point-of-care applications [27,28].

In this study, a portable detection device was developed, designated the **C**RISPR-based **c**ompact **a**utomated **r**eal-time **d**etection (Ccard), comprising a microfluidic chip and a miniaturized device. The device has been engineered to facilitate rapid and efficient detection of HPV16, HPV18, and the internal reference gene GAPDH. In comparison with existing CRISPR-Dx technologies, the Ccard system significantly simplifies sample processing, dispensing, and CRISPR detection analysis workflows. Initially, user-collected cervical swab samples undergo viral lysis at ambient temperature. Subsequently, the samples undergo automated passive metering via physical PTFE blocking, being precisely distributed into four detection wells under negative pressure. Secondly, the system employs a single-step RPA-CRISPR detection, which streamlines the entire assay by integrating amplification and detection in a single reaction. Furthermore, the Ccard system incorporates detection of a human internal reference gene to ensure the reliability of the results. Finally, the results of the detection process are transmitted wirelessly to smartphones via Bluetooth Low Energy (BLE) technology, thereby enabling real-time visualization of fluorescence curves. The results reveal that the Ccard system possesses the capability to detect HPV16 and HPV18 in approximately 30 min, with a detection limit (LOD) as low as 5 copies/μL. In summary, the Ccard system significantly simplifies the HPV testing workflow with minimal, pipette-based manual operation, showing strong potential for rapid HPV detection in resource-limited settings.

## 2. Experimental Section

### 2.1. Chemicals and Reagents

All chemical reagents and reagents used in this study were of analytical grade or higher purity, as listed in Supplementary Table S1.

### 2.2. Target DNA Preparation

Human genome and HPV 16, 18 DNA quantitative reference standards are provided by BeNa Culture Collection (Suzhou, China). Graded DNA reference standards are diluted and stored at −20 °C until use. The quality controls of HPV31, HPV33, HPV35, HPV39, HPV45, HPV51, HPV52, HPV56, HPV58, HPV59, HPV66, and HPV68 gDNA were purchased from GeneWell Technology, Co., Ltd. (Shenzhen, China).

### 2.3. Viral Lysis

A negative clinical cervicovaginal swab sample was utilized to optimize and verify the effectiveness of the lysis method. Specifically, a commercial room-temperature sample release agent (GenDx Biotech, Suzhou, China, Cat. no. GD005) was added to the cervicovaginal swab sample at an optimized volume ratio of 1:1 (*v*/*v*). Then, the tube was capped, inverted, and mixed 8–10 times, incubated at room temperature for 5 min to release nucleic acid, and then proceeded with the single-step RPA-Cas13 CRISPR assay for GAPDH gene detection. The released lysate was used directly in the downstream reaction without any purification, neutralization, or removal step. Then, the lysis efficacies under various lysis conditions, including volume ratios of lysis buffer to sample and volume of lysed sample, were evaluated. In addition, the DNA from the same negative clinical cervicovaginal swab sample was extracted using TIANamp Genomic DNA kit (TIANGEN Biotech, Beijing, China) as a positive control.

### 2.4. Design of RPA Primers and crRNAs

The highly conserved region of the HPV L1 gene was selected as the specific detection target for HPV18. RPA primers and crRNA sequences were designed using Primer Premier 5 and NCBI BLAST2.14.0 tools. The RPA primer design followed the guidelines outlined in the TwistAmp detection manual, while the crRNA was optimized based on the general design principles for the Cas13a system. All primers and crRNAs were synthesized by Sangon Biotech (Shanghai, China).

### 2.5. Single-Step RPA-Cas13a Assay

Single-step RPA-CRISPR/Cas13a assay was established for HPV16, HPV 18 and GAPDH detection. The optimized reaction consisted of 90 µL master mix: 1× rehydration buffer, 1 U/µL RNase inhibitor, 0.5 mM rNTP mix, 1 U/µL T7 RNA polymerase, 250 nM forward primer, 250 nM reverse primer, 25 nM crRNA, 1 µM FAM-UUUUU-BHQ1, and 50 nM LwaCas13a. Then, the prepared master mix was added to resuspend the RPA-lyophilized pellet and divided into five 18 µL reaction units. Subsequently, the reaction was activated through the addition of 1 µL DNA target and 1 µL 280 mM magnesium acetate to the reaction mixture. Finally, the 20 µL reaction system was incubated at 37 °C for 30 min using a fluorescence plate reader (ABI-7500, Applied Biosystems, Foster City, CA, USA), and the fluorescence curves were monitored in real time.

### 2.6. Preparation of Lyophilized Reagent

Lyophilized reagents were prepared from a 30 µL pre-mix. Since each chamber was reconstituted to a final volume of 8 µL before use (Section 2.9), the pre-mix was formulated to give the following working concentrations in that 8 µL reaction: 1 U/µL RNase inhibitor, 0.5 mM ribonucleoside triphosphate (rNTP) mix, 1 U/µL T7 RNA polymerase, 250 nM forward primer, 250 nM reverse primer, 25 nM crRNA, 1 µM FAM-UUUUU-BHQ1, and 50 nM LwaCas13a. The pre-mix also contained 1× lyophilization buffer, prepared by diluting a 5× stock of 10% trehalose, 1% mannitol, and 1% polyethylene glycol-20000. The stock concentration of each reagent, the volume added to the 30 µL pre-mix, and the amount lyophilized per chamber are listed in Supplementary Table S2.

After vortexing, the pre-mix was used to fully resuspend one tube of commercial RPA lyophilized powder (TABAS03KIT, TwistDx, Maidenhead, UK). Aliquots of 2.1 µL were dispensed into the reaction wells of the microfluidic chip, and the chip was placed in a lyophilizer (Labconco, Kansas City, MO, USA). The reagents were pre-cooled at −60 °C for 1–2 h to ensure complete crystallization and then dried under high vacuum for 8–10 h, so that the RPA enzyme and the pre-mixed reagents were co-lyophilized within each chamber. The lyophilized chips were stored at room temperature until use.

### 2.7. Fabrication of the Microfluidic Chip

Polymethyl methacrylate (PMMA) was selected as the substrate material for the microfluidic chip due to its exceptional optical transparency, heat resistance, and ease of processing. The dimensions of the chip were designed as 65 mm in length, 22 mm in width, and 3 mm in thickness using 3D CAD software (SolidWorks 2023, USA). The fabrication process is outlined as follows: Initially, a HY-6090 CNC engraving machine(Huayue, Jinan, China) was employed to meticulously carve microchannel structures onto transparent PMMA plates. During the engraving process, strict control of process parameters was maintained to ensure that the channel surfaces were smooth and free of burrs, while maintaining high flatness for successful bonding with pressure-sensitive adhesive (PSA). Subsequently, the fabricated substrates were subjected to an ultrasonic cleaning procedure. The protocol involved a series of steps, including ultrasonic cleaning in 95% ethanol at room temperature for five minutes to remove organic contaminants. This was followed by ultrasonic cleaning in deionized water at 50 °C for 15 min to eliminate residual impurities. Subsequently, the substrates were placed in a vacuum drying oven and dried at 80 °C for 30 min to ensure the complete removal of moisture and solvent residues, thereby yielding clean and dry substrates. The key dimensions of the microfluidic features, including channel widths, chamber depth and volume, inlet reservoir, air outlet, and pressure-relief hole sizes, as well as the material specifications of the pressure-sensitive adhesive and PTFE membrane, are summarized in Supplementary Table S3 and illustrated in Figure S1.

### 2.8. Fabrication of the Portable Device

The portable Ccard device, with dimensions of 108 mm in length, 82 mm in width, and 73 mm in height, and 320 g in weight, is a Type-C powered device. The device has been engineered to function in conjunction with a complementary microfluidic chip, which is employed for the detection of HPV16, HPV18, and GAPDH genes. The final design of Ccard was created using three-dimensional computer-aided design (CAD) software (SolidWorks 2023, USA) and was produced with a stereolithography (SLA) 3D printer (Union, Shanghai, China) with a manufacturing accuracy of 0.2 mm. The outer shell of the device is coated with a light-blocking paint to prevent external light interference, ensuring precise real-time fluorescence signal collection.

The control circuit of the device was designed using EasyEDA2.2.45 for both the schematic and PCB layout. The power supply module employs a standard Type-C interface, compatible with prevalent power delivery (PD) protocol (9V/3A) chargers. The microcontroller unit is based on the low-power ESP32-S3 series processor (Espressif, Shanghai, China). On the PlatformIO platform, the system is developed based on the Arduino2.2.1 framework and lightweight FreeRTOS 10.5.1 operating system, enabling multi-task non-blocking operation to meet the needs of complex task scheduling. The temperature control system utilizes an incremental proportional–integral–derivative (PID) strategy. A high-precision 24-bit analog-to-digital converter (AD7793, Analog Devices, Norwood, MA, USA) samples temperature data from a Negative Temperature Coefficient thermistor at 20 Hz for real-time feedback. Subsequently, the system adjusts the Pulse Width Modulation (PWM) duty cycle to regulate the heating power of the Polyimide film by controlling the Metal-Oxide-Semiconductor Field-Effect Transistor (MOSFET) switch circuit. The fluorescence acquisition system is composed of excitation and detection units. The fluorescent probe used in this study is FAM, with an excitation wavelength of approximately 490 nm and emission at about 520 nm. The excitation is provided by a 470 nm LED, whose brightness is precisely regulated by the multi-channel Low Dropout Regulator (LDO)-based constant current source. The excitation light is focused onto the reaction system within the well through a bandpass filter. The AS7341 spectral sensor (AMS Austria, Premstätten, Austria) is utilized for the capture of fluorescence signals due to its affordability, compact size, and high sensitivity. Due to the hardware limitations of the main control chip’s Inter-Integrated Circuit (I2C) interface, a software-based I2C timing method is employed to expand communication, thereby allowing multiple AS7341 sensors to operate collaboratively. The fluid movement system is controlled by the MCU, which activates the MOSFET electronic switch to regulate the negative pressure pump. The facilitation of human–machine interaction is enabled by Bluetooth Low Energy (BLE) communication. The mobile application has been developed using the Flutter2.18.0 framework, which facilitates the creation of cross-platform applications (e.g., Android, iOS, etc.) with modern interfaces.

### 2.9. Detection Operating Procedure

When utilizing Ccard, it is imperative to incorporate 25.6 μL of rehydration buffer (consisting of 23.6 μL of RPA rehydration buffer and 2 μL of 280 mM magnesium acetate) along with 14.4 μL of the target sample into the sample port of the microfluidic chip (40 µL total). Here, the 14.4 μL target sample refers to the crude lysate, i.e., the cervicovaginal swab sample pre-mixed 1:1 (*v*/*v*) with the sample release agent; the release agent is not removed and participates directly in the reaction. After the dispensing of the reagents, the lyophilized reagent will undergo resuspension and activation to initiate the reaction, followed by the single-step RPA-CRISPR/Cas13a reaction at 37 °C for 30 min.

### 2.10. qPCR Assay

The assay protocol of qPCR is as follows: Premix Ex Taq (Probe qPCR, Takara Bio Inc., Otsu, Japan) was used to set up qPCR detection for HPV16, HPV18, and GAPDH. Each 25 μL reaction mixture contained 12.5 μL of 2× Premix Ex Taq (Probe qPCR), 400 nM of forward and reverse primers, 400 nM of FAM probe, 2 μL of DNA template, and 7.5 μL of nuclease-free water. The thermal cycling program included the following steps: 50 °C for 2 min, 95 °C for 2.5 min, followed by 45 cycles of 94 °C for 30 s and 55 °C for 30 s.

### 2.11. Analysis of Clinical Samples

The study received approval from the Clinical Research Ethics Committee of the Second Affiliated Hospital of Henan University of Traditional Chinese Medicine (PZ-HNSZYY-2022-008). A total of 50 clinical samples were collected from the hospital under consideration. A cross-sectional analysis was then conducted to evaluate the effectiveness of the Ccard test in detecting HPV16 and HPV18, and to compare its performance with that of the qPCR method. All the samples were stored at −20 °C until use.

### 2.12. Statistical Analysis

Statistical analysis was performed using GraphPad Prism 10. A two-tailed Student’s *t*-test was used to analyze the data. Statistical significance was considered at a *p*-value < 0.05. To set the cutoff value, we detected 20 no-target controls using Ccard. The positive threshold was defined as the mean endpoint fluorescence of the negative controls plus four standard deviations (*n* = 20 no-template control replicates, measured at 30 min). This cutoff was established prior to clinical sample analysis.

## 3. Results and Discussion

### 3.1. Overview of the Ccard

**Figure 1:**
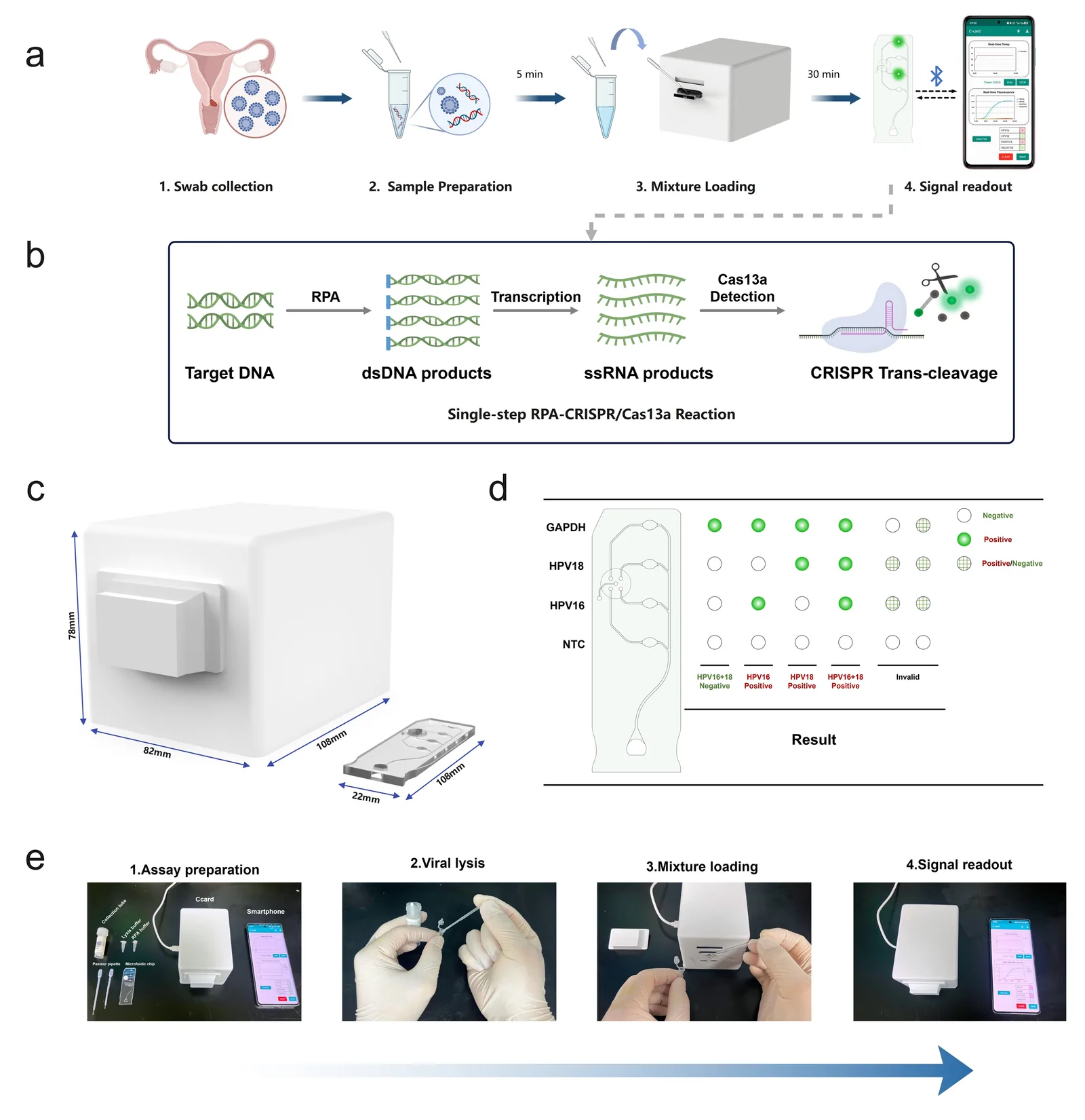
Overview of the CRISPR-based Compact Automated Real-time Detection (Ccard) system for rapid testing of HPV16 and HPV18. (**a**,**b**) Schematic diagram illustrating the Ccard workflow for HPV virus detection. Collected cervical samples undergo a 5 min extraction-free viral lysis at room temperature. The released HPV viral DNA is processed at 37 °C for 30 min using a single-step RPA-CRISPR/Cas13a reaction. Test results are analyzed via a wireless-connected smartphone application. (Created with bioRender.com). (**c**) Dimensions of the Ccard device. (**d**) Result interpretation rules displayed by app indicators. (**e**) Operational workflow of the Ccard for HPV detection. Created in BioRender. lj, Y. (2026) https://BioRender.com/ok430lt.

In this study, we developed an integrated microfluidic CRISPR-based diagnostic device, termed Ccard, enabling rapid, user-friendly, and simultaneous detection of HPV16, HPV18, and the internal control gene GAPDH. As illustrated in Figure 1a, cervical samples were collected via cervical swabs and subsequently subjected to a 5 min extraction-free viral lysis at room temperature. The lysate was then mixed with RPA buffer and magnesium ions, and the mixture was loaded into the inlet of a microfluidic chip. Notably, while negative-pressure-based loading has been previously explored, the Ccard platform features a minimalist fluidic architecture and synergistic co-design of hardware and biochemistry. Unlike established platforms requiring complex multi-layer fabrication or active components like wax valves (Supplementary Table S4), Ccard leverages a single passive PTFE membrane as a “physical logic gate” to achieve autonomous self-stopping and precise volumetric metering without active valves. Driven by an integrated vacuum pump, the mixture flows into the reaction chambers through a passive physical blocking mechanism, ensuring consistent rehydration of the lyophilized RPA-CRISPR/Cas13a reagents while achieving precise fluidic termination. This was followed by a 30 min isothermal incubation to carry out the single-step RPA-CRISPR/Cas13a reaction. Real-time fluorescence signals generated during the reaction were wirelessly transferred to a smartphone via Bluetooth. The single-step RPA-CRISPR/Cas13a reaction integrates RPA, T7 transcription, and CRISPR/Cas13a detection within a reaction well, which could avoid aerosol contamination and further simplify the workflow (Figure 1b).

The complete workflow of the single-step RPA–CRISPR/Cas13a assay, including liquid loading, isothermal incubation, real-time fluorescence acquisition, and automated result interpretation, is seamlessly integrated into a miniaturized device, termed Ccard (Figure 1c). The Ccard features a smartphone interface that displays real-time fluorescence curves and automatically interprets detection results, with the interpretation logic illustrated in Figure 1d.

The detection system is composed of an EP tube containing lysis buffer, an EP tube containing RPA buffer and magnesium ions, two Pasteur pipettes (15 μL and 50 μL), a microfluidic chip, a dedicated device, and a smartphone for wireless control. The procedure entails the quantitative transfer of the sample from the collection tube to an EP tube containing lysis buffer using a Pasteur pipette with a capacity of 15 μL. This process initiates the release of nucleic acids. Subsequently, the nucleic acid-released sample is transferred into an EP tube containing RPA buffer and magnesium ions using a Pasteur pipette with a capacity of 15 μL again. Subsequently, the mixed reagents are transferred via a Pasteur pipette (50 μL) to the inlet port of the microfluidic chip, which has been previously inserted into the Ccard device. Upon pressing the “Run” button in the application, the mixture is automatically loaded into the reaction wells under negative pressure generated by an integrated vacuum pump. Subsequent to the dispensing process, plastic patches are meticulously affixed over the inlet port. Subsequently, the Ccard automatically performs temperature control and fluorescence detection in the reaction area (Figure 1e). Ccard is capable of identifying two high-risk subtypes, HPV16 and HPV18, in approximately 30 min under resource-limited conditions, showing promise for decentralized HPV screening. Notably, while sample lysis and loading remain manual pipette-based steps, the downstream negative-pressure loading, passive volumetric metering, temperature control and real-time detection are automated and integrated on the chip.

### 3.2. Development and Optimization of Single-Step RPA-CRISPR/Cas13a Assay

**Figure 2:**
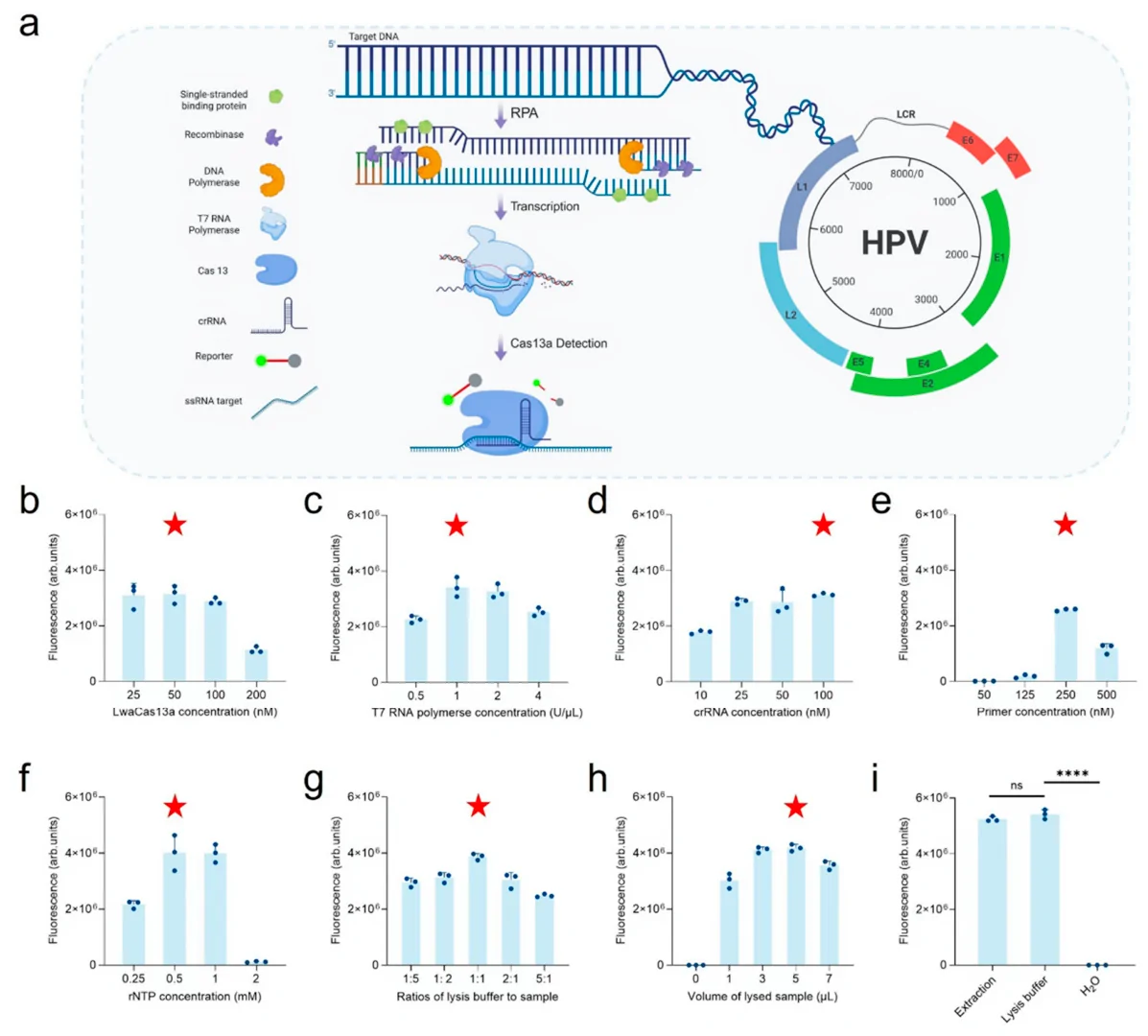
Optimization of key components in the single-step RPA-CRISPR/Cas13a detection system. (**a**) Schematic representation of the single-step RPA-CRISPR reaction. Endpoint fluorescence intensity is measured to optimize six critical reaction conditions (Created with bioRender.com). The fluorescence intensity at 10 min of the single-step RPA-Cas13a reaction is measured for the optimization of reaction conditions. (**b**) Primer concentrations were tested at 50, 125, 250, and 500 nM. (**c**) LwaCas13a concentrations were tested at 25, 50, 100, and 200 nM. (**d**) crRNA concentrations were tested at 10, 25, 50, and 100 nM. (**e**) T7 RNA polymerase concentrations were tested at 0.5, 1, 2, and 4 U/μL. (**f**) rNTP concentrations were tested at 0.25, 0.5, 1, and 2 mM. (**g**) The ratio of lysis buffer to sample was tested at 1:5, 1:2, 1:1, 2:1, and 5:1. (**h**) Volumes of lysed sample were tested at 0, 1, 3, 5, and 7 μL. (**i**) Endpoint fluorescence intensity comparison showed that direct lysis buffer samples yielded a signal comparable to extracted DNA, both significantly higher than the negative control (two-tailed Student’s *t*-test; **** denotes *p* < 0.0001), demonstrating the feasibility of direct detection without DNA extraction. All key parameters marked with red stars in the figures were optimized accordingly. Created in BioRender. lj, Y. (2026) https://BioRender.com/9ednerb.

This study presents a novel integrated detection platform that enables rapid, simple, and highly sensitive nucleic acid testing for HR-HPV by combining RPA, in vitro transcription, and CRISPR/Cas13a detection within a single reaction system. Under isothermal conditions, the RPA enzyme system (including recombinase, single-stranded binding protein, and DNA polymerase) was employed to amplify the target DNA (the L1 region of the HPV genome), generating double-stranded DNA (dsDNA) products. To achieve signal conversion, a T7 promoter sequence was designed at the 5′ end of the forward primer, enabling the amplified product to be recognized by T7 RNA polymerase for transcription into single-stranded RNA (ssRNA). The generated ssRNA was specifically recognized by the Cas13a-crRNA complex, where the crRNA binds to the target ssRNA through base pairing, thereby activating the non-specific trans-cleavage activity of Cas13a. The activated Cas13a randomly cleaves a fluorescently labeled reporter molecule (5′-FAM-UUUUU-BHQ1-3′) within the reaction system. This reporter molecule carries an FAM fluorophore and a BHQ1 quencher, and upon cleavage, it releases a significant fluorescence signal, allowing real-time monitoring of the detection results (Figure 2a). To highlight the structural advantages of this integrated design, we performed a systematic comparison with existing CRISPR-based detection methods (Supplementary Table S5). Unlike traditional two-step assays (e.g., SHERLOCK, DETECTR) or lateral flow approaches which are susceptible to aerosol contamination and typically require turnaround times exceeding 60 min, our closed-tube, single-step system effectively minimizes contamination risks and significantly accelerates the workflow to approximately 30 min.

After confirming conservation through multiple sequence alignments, systematic screening identified the best primer-probe combination as F1/R3/crRNA3 (Figure S2). The primers and crRNAs for HPV16 and GAPDH were based on our previous work. The sequences of the targeted HPV16, HPV18, and GAPDH are listed in Supplementary Table S6. The sequences of primers and CRISPR crRNAs are provided in Supplementary Table S7. To maximize detection sensitivity, a systematic optimization of the parameters of each component in the reaction system was performed to determine the optimal concentrations (Figure 2b–f and Figure S3). The fluorescence intensity measured at the 10 min time point of the single-step RPA–Cas13a assay for HPV18 detection was used as the evaluation metric. The optimization results indicate that the system achieved optimal performance when the primer concentration was 250 nM, LwaCas13a concentration was 50 nM, crRNA concentration was 25 nM, T7 RNA polymerase concentration was 1 U/μL, and rNTP mixture concentration was 0.5 mM.

In parallel, we optimized the sample preparation workflow. The fluorescence intensity measured at the 10 min time point of the single-step RPA–Cas13a assay for GAPDH gene detection was used as the evaluation metric. In a 20 μL single-step CRISPR detection system, an optimized 1:1 lysis ratio of buffer to sample was used, with 5 μL of lysate input after 5 min of room-temperature lysis. This optimization strategy simplifies operational procedures while maintaining optimal detection performance compared with traditional nucleic acid extraction methods (Figure 2g–i).

### 3.3. Design and Principle of the Ccard

The Ccard system is composed of three fundamental components: a microfluidic chip facilitating the single-step RPA-CRISPR reaction; a customized peripheral device positioned around the chip; and a smartphone application that enables human–machine interaction.

**Figure 3:**
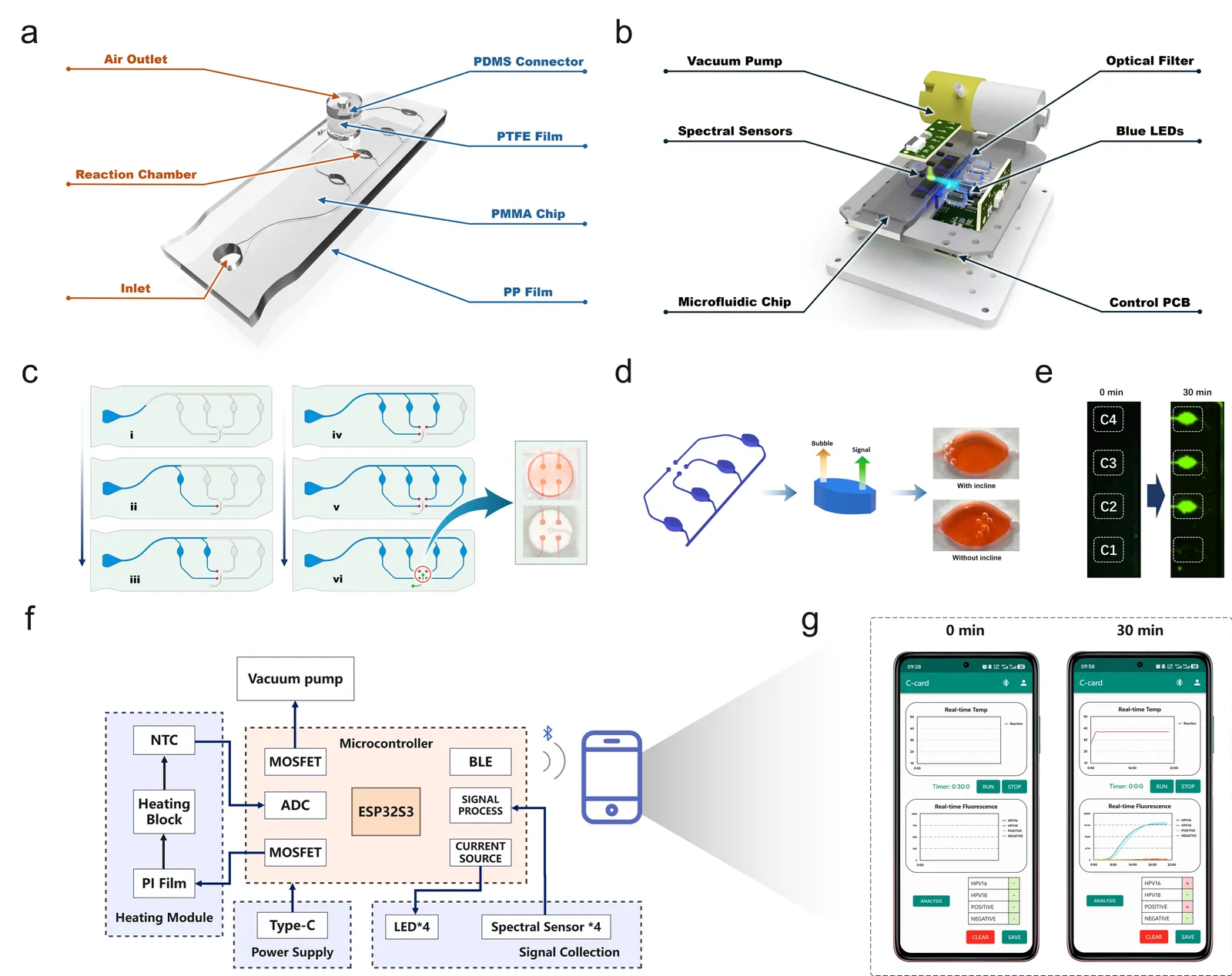
Fabrication of the portable analysis device. (**a**) Exploded view of the microfluidic chip. The right side shows the chip structure, while the left side highlights its functions. (**b**) Exploded view of the Ccard device. (**c**) Schematic diagram of fluid sequential entry and results with and without pressure relief holes. (**d**) Anti-bubble tilted design of chip reaction wells. (**e**) Fluorescence imaging of chips before and after the reaction. (**f**) Schematic diagram of the Ccard device. (**g**) The software interface displays before and after the reaction.

The microfluidic chip’s functional zones include the inlet, the reaction chambers, and the air outlet (Figure 3a). The microfluidic chip and its internal reaction chambers were fabricated via CNC machining with a structural tolerance of ±0.1 mm to ensure volumetric consistency across the four reaction chambers. The procedure entails the injection of a mixture comprising sample-released nucleic acids, RPA buffer, and magnesium ions into the sample inlet. After this step, the solution is drawn into the reaction chambers under negative pressure. Each reaction well has a capacity of 8 μL of solution. The 40 µL loaded volume is sufficient to completely fill the four reaction chambers while leaving a residual volume in the upstream channel to prevent contamination; a complete liquid-volume balance of the chip (chamber, channel, inlet reservoir and dead volumes) is provided in Supplementary Table S8. This compact apparatus guarantees expeditious and consistent heating of the reaction system (Figure S4). Driven by the vacuum pump, the sample moves along the microchannel until it reaches the periphery of the PTFE membrane. The membrane’s microporous structure accounts for its hydrophobicity, which prevents liquid permeation. Despite its hydrophobicity and breathability, Laplace equation analysis indicates the presence of a critical pressure threshold under specific conditions [29,30].

$$\Delta P_{c}=\frac{2\sigma \cos\theta }{r}$$

Δ*P_c_* is equivalent to the critical pressure (Pa); *σ* is the surface tension of the reaction system (N/m); *θ* is the contact angle of the reaction system on the polytetrafluoroethylene (PTFE) surface (°); and *r* is the equivalent radius of the membrane pore (m).

In order to prevent liquid breakthrough through the PTFE membrane during negative pressure extraction, vent holes are incorporated into the design with the specific purpose of relieving pressure and ensuring system stability (Figure 3c). The chip is connected to external devices via a polydimethylsiloxane (PDMS) connector, which features excellent sealing performance, efficient negative pressure transmission capability, and robust elasticity, enabling tight contact with the heating block. This approach enhances system integration, ensures effective heat transfer, and improves the chip’s thermal management capabilities (Figure S5). The reaction wells are designed as elongated spindle shapes to guarantee thorough resuspension of lyophilized reagents within the chamber. The inclination of the inner walls serves to direct the bubbles generated during suspension and reaction toward the opposite side of the detection port (Figure 3d). The fishbone-shaped chip design, coupled with an ultra-narrow chamber channel width of 0.3 mm from front to back, effectively prevents cross-contamination between adjacent reaction chambers, as confirmed by ImageJ1.54k quantification of inter-chamber fluorescence signals (mean gray value: positive chambers of 252 vs. C1 blank chamber of 19; Figure 3e and Figure S6). To demonstrate the system’s reliability when handling challenging clinical samples, we tested the device with a high-viscosity simulant (40% *v*/*v* glycerol solution, ~4.8 mPa·s). This viscosity is higher than the typical physiological range of human whole blood (3.5–4.5 mPa·s), which is widely recognized as a benchmark for high-viscosity biological fluids in microfluidic studies [31]. The PTFE-mediated physical interface successfully terminates the flow, demonstrating a robust stopping mechanism (Figure S7).

The device, powered by Type-C, consists of three functional modules (Figure 3b,f and Figure S8). First, the subsystem of fluid control comprises the chip outlet sealing structure and a vacuum pump, which is utilized to generate negative pressure. Upon insertion of the chip with a PDMS soft connector into the device, the connector gradually forms a tight seal between the internal fluid cavity of the chip and the external negative pressure system through its tilted internal design (Figure S5), ensuring smooth sample pipetting. Second, the subsystem of temperature control comprises two closed-loop components, namely, heating and temperature sensing, to ensure precise thermal management of the microfluidic chip (Figure S9). The heating component employs a 6063 aluminum alloy substrate for the heating block, with the objective of enhancing heat distribution uniformity. The underside of the heating block is coated with a back-adhesive polyimide (PI) film, which provides approximately 3 W of power output. The temperature sensing assembly employs a high-precision negative temperature coefficient (NTC) thermistor, which is fixed within the heating block using thermal paste. Lastly, the real-time fluorescence detection system is composed of two units: an excitation unit and a detection unit. The excitation unit, which comprises a 470 nm LED with 130 mW of power and a 480 nm filter, works in tandem with the detection unit that employs four AS7341 spectral sensors, each capable of multi-channel detection, and in this configuration, the F4 channel (505–525 nm) is utilized to collect fluorescence data in real-time, effectively replacing conventional and often costly filter-based optical systems [32,33]. The excitation and detection components are arranged orthogonally to minimize the impact of excitation source background signals on detection results. The sensor is positioned 1 mm away from the microfluidic chip, enabling detection of FAM fluorescence concentrations as low as 0.1 μM (Figure S10). Concurrently, the heating block under the chip, which has been anodized to a matte black finish, serves to mitigate the incidence of scattered light contamination within the fluorescence detection path (Figure 3a). As demonstrated in Supplementary Table S9, the cost of the integrated device, comprising mechanical components, a microfluidic chip, and electronic elements (Figures S11–S13), is less than $34, making it a cost-effective solution for environments with limited resources.

A smartphone application has been developed to facilitate the control of the device via a Bluetooth wireless connection (Figure 3f,g). The system is capable of real-time monitoring of temperature and fluorescence changes. Additionally, it possesses the capacity to automatically analyze and interpret fluorescence signals. Furthermore, the system has the capacity to support multiple users and manage data concurrently (Figure S14).

### 3.4. Analytical Performance of Ccard for HPV16 and HPV18 Detection

**Figure 4:**
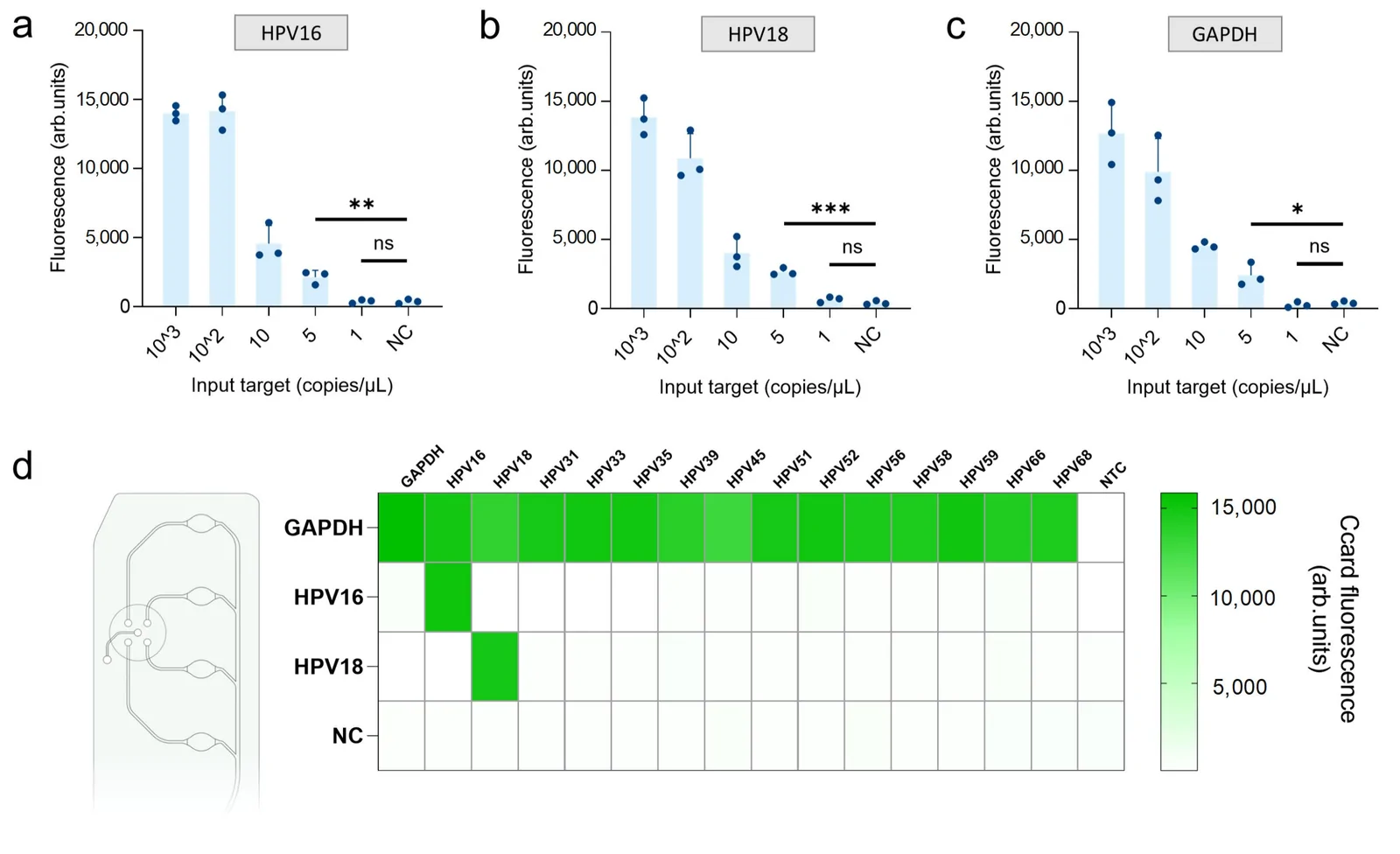
Evaluation of the analytical performance of the Ccard system. (**a**) Sensitivity of HPV16 detection on the chip. (**b**) Sensitivity of HPV18 detection on the chip. (**c**) Sensitivity of GAPDH detection on the chip. (**d**) Specificity of primer sets for HPV16, HPV18, and GAPDH on the chip. *p*-values were calculated by two-tailed Student’s *t*-test. *** means *p* < 0.001. ** means *p* < 0.01. * means *p* < 0.05. ‘ns’ means no significance, *p* > 0.05.

Subsequently, a systematic evaluation was conducted to assess the performance of the Ccard system in detecting HPV. In the experiment, a series of gradient concentrations (10^3^ to 1 copy/μL) of nucleic acid standards was utilized to perform the single-step RPA-CRISPR reaction on the detection chip to determine the limit of detection (LOD). Real-time fluorescence curve analysis results (Figure S15) demonstrated that the LODs for HPV16, HPV18, and GAPDH were all 5 copies/μL (*n* = 3) (Figure 4a–c).

A reaction was scored as positive when its endpoint fluorescence at 30 min exceeded a fixed threshold. The threshold was defined as the mean endpoint fluorescence of the negative controls plus four standard deviations (n = 20 no-template controls, measured at 30 min; mean = 414.46, SD = 197.00; calculated value = 1202.46), and was conservatively set at 1500 to further minimize the risk of false positives. This cutoff was established before the clinical samples were analyzed.

A positive call is only meaningful if the reaction stays dark without target, so we next tested six negative-control reactions on the chip: no target template (NTC), no primers, no crRNA, no Cas13a, no T7 RNA polymerase, and reporter only. At 30 min, none of the six controls exceeded the threshold of 1500, whereas the complete reaction containing 5 copies/µL HPV16 reached approximately 3000 (Figure S16). The NTC was prepared in lysis buffer and therefore also served as the lysis-buffer blank, ruling out background from the lysis reagent.

To assess whether components of the cervical-swab matrix interfere with the assay, we further evaluated the analytical sensitivity directly in negative cervicovaginal swab lysate. Serial concentrations of HPV16 and HPV18 targets (10, 5, 1 copies/µL and NTC) were spiked into negative swab lysate and compared with the same concentrations prepared in buffer (Figure S17). In the lysate matrix, both HPV16 and HPV18 were reliably detected down to 5 copies/µL (Figure S17a,b), consistent with the LOD obtained using purified reference DNA. Side-by-side comparison of lysate versus buffer (Figure S17c,d) showed that the swab matrix caused only a modest reduction in endpoint fluorescence at higher target concentrations, whereas the signal at 5 copies/µL remained clearly above the NTC and the detection limit was unchanged. These results indicate that the cell debris and proteins released during lysis do not inhibit amplification or compromise the detection performance of Ccard.

To validate the specificity of the system, a series of tests was conducted using nucleic acid standards containing multiple HPV genotypes (HPV16, HPV18, HPV31, HPV33, HPV35, HPV39, HPV45, HPV51, HPV52, HPV56, HPV58, HPV59, HPV66, and HPV68) and human genomic DNA standards (n = 3). Experimental data (Figure 4d) and fluorescence imaging (Figure 3e) analyses reveal that the Ccard achieves remarkable specificity by leveraging a dual-assurance mechanism with a novel design that integrates a fishbone-shaped microfluidic chip. This mechanism integrates RPA primer-guided specific amplification with CRISPR crRNA-mediated targeted recognition, forming a dual safeguard to ensure the accuracy and reliability of detection results. Furthermore, several measures have been implemented to prevent cross-contamination between the microfluidic chip and external devices. This process involves the implementation of plastic patches at the chip inlet during each reaction, as well as the integration of two layers of PTFE membrane at the outlet. The PTFE membrane, with its exceptional aerosol and liquid barrier properties, effectively prevents contamination between the chip, external devices, and reaction zones [34,35]. In order to assess the storage stability of the lyophilized reagents on the chip, the chips were stored at both 4 °C and room temperature for a period of one month before testing. The results (Figure S18) demonstrate that the enzyme activity of the lyophilized reagents remained stable during storage, indicating their exceptional long-term stability [36].

Compared with existing HPV detection technologies, the Ccard platform demonstrates a strategic balance of performance, safety, and portability. The sample processing protocol is streamlined to just 5 min at ambient temperature, with a manual workflow simplified to Pasteur pipette-based operations. Despite utilizing this extraction-free direct lysis protocol, the system achieves a competitive LOD of 5 copies/μL for HPV16 and HPV18 within 30 min while maintaining excellent specificity (Supplementary Table S10). Furthermore, a comparison with recently published microfluidic and CRISPR platforms (Supplementary Table S4) confirms that the Ccard offers superior sensitivity over paper-based devices and eliminates the need for bulky equipment to ensure a truly handheld form factor (320 g). Combined with real-time smartphone monitoring, this platform provides a robust solution for rapid testing in resource-limited settings [15,37,38,39].

### 3.5. Validation of Clinical Samples

**Figure 5:**
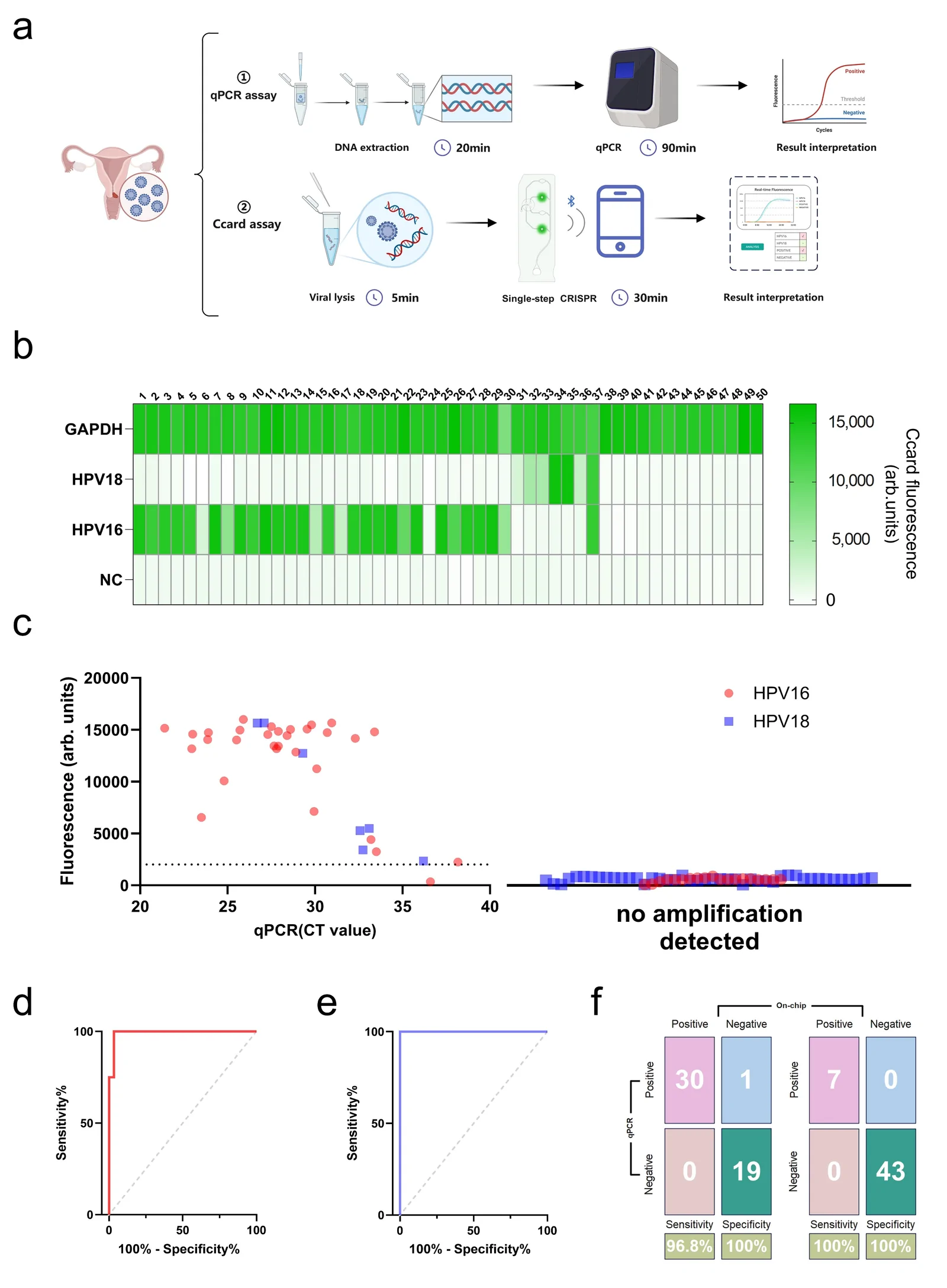
Validation of the clinical feasibility of the Ccard system. (**a**) Workflows comparison between qPCR assays and the single-step duplex CRISPR assay for testing clinical cervicovaginal swab samples. (**b**) Analysis of 50 cervical swab samples using the Ccard. The heatmap displays endpoint fluorescence values of the test results. (**c**) Correlation between qPCR Ct values and fluorescence intensity readouts by Ccard in clinical samples. (**d**) ROC curve for HPV16 detection in clinical samples from the Ccard platform. The area under the curve (AUC) for the HPV16 detection assay was 0.992. (**e**) ROC curve for HPV18 detection in clinical samples from the Ccard platform. The AUC for the HPV18 detection assay was 1.000. (**f**) Confusion matrix summarizing the performance of the Ccard in detecting HPV clinical samples (left: HPV16; right: HPV18).

The conventional qPCR workflow necessitates a 20 min duration for the extraction of viral nucleic acids, followed by 90 min for the detection. Conversely, the Ccard method necessitates a mere 5 min of viral lysis at ambient temperature, subsequently followed by a 30 min four-channel single-step RPA-CRISPR reaction for the detection process (Figure 5a). The present study encompassed the analysis of 50 clinical samples (Figure 5b): 30 samples were HPV16 single-positive, 6 were HPV18 single-positive, 1 was co-infected with both HPV16 and HPV18, and 13 served as negative controls; accordingly, 31 samples were HPV16-positive and 7 were HPV18-positive. The clinical utility of the Ccard detection system was evaluated in parallel with quantitative PCR (qPCR) (Figures S19 and S20). Reaction chambers C2, C3, and C4 were preloaded with lyophilized detection reagents targeting HPV16, HPV18, and GAPDH, respectively, while C1 served as a blank control containing all reaction reagents except the primers and crRNA, used to monitor optical background. All clinical samples were scored using the positive threshold of 1500 defined in Section 3.4. As shown in Figure 5b,c, the system successfully detected 29 of the 30 HPV16 single-positive samples. The single undetected sample had a Ct value of 36.6, while another sample with an even higher Ct value (38.2) yielded a positive signal, likely reflecting stochastic variation in target availability. The HPV16/HPV18 co-infected sample was detected in both channels and was therefore counted as a true positive in each channel analysis. Overall, the system exhibited slightly lower sensitivity compared with conventional qPCR. This difference can be attributed to the chip’s architecture: each sample is distributed across four independent reaction chambers, resulting in an approximate fourfold dilution of the original nucleic acid concentration. Furthermore, the reduced reaction volume per well which is smaller than that used in standard qPCR also contributes to the modest decrease in detection sensitivity. For HPV18, seven samples were positive (six HPV18 single-positive and one HPV16/HPV18 co-infected case; the co-infected sample was counted as a true positive in each respective channel analysis) and 43 were negative; all were correctly identified. Concurrently, all 13 negative samples were accurately identified. ROC curve analysis revealed AUC values of 0.992 for HPV16 and 1.000 for HPV18 (Figure 5d,e). The Ccard system achieved a sensitivity of 96.8% (30/31; 95% CI: 83.3–99.9%) and specificity of 100% (19/19; 95% CI 82.4–100%) for HPV16, and 100% sensitivity (7/7; 95% CI: 59.0–100%) and 100% specificity (43/43; 95% CI: 91.8–100%) for HPV18 (Figure 5f). The findings demonstrate that this testing platform offers advantages in the streamlining of workflows, the reduction in turnaround times, and the facilitation of easy deployment. The assay’s ability to concurrently detect HPV16 and HPV18 is particularly noteworthy, especially in resource-limited settings. It should be noted that the number of HPV18-positive samples in this cohort was limited (n = 7); multi-center studies with larger cohorts are warranted to consolidate these diagnostic performance estimates.

## 4. Conclusions

In summary, this study developed a Ccard platform featuring a streamlined workflow that couples a 5 min extraction-free lysis with automated chip-based CRISPR detection. While the current sample lysis and loading are performed externally via a simple pipette-based operation, the downstream processes, including passive metering via physical PTFE blocking and automated distribution into multiple reaction chambers, are integrated on the microfluidic chip. Driven by a portable vacuum pump, the system demonstrates reliable performance, achieving concurrent detection of HPV16, HPV18, and GAPDH within 30 min, with LODs as low as 5 copies/µL and the ability to detect clinical samples with Ct values up to 38.2. The clinical evaluation demonstrated a 96.8% sensitivity for HPV16 and a 100% sensitivity for HPV18, with 100% specificity for both targets. To transition towards a truly sample-in-answer POCT device, future research will focus on integrating a dedicated sample-preprocessing module (such as a lysis chamber with pre-filled reagents or a filtration membrane) directly onto the Ccard. This would eliminate manual liquid handling and allow for the direct processing of clinical cervical swabs, further enhancing the system’s utility in resource-limited settings.

## Data Availability

The data presented in this study are available on request from the corresponding authors.

## Supplementary Materials

The following supporting information can be downloaded at: https://www.mdpi.com/article/10.3390/bios16100571/s1, Figure S1: Schematic diagram of the Ccard microfluidic chip; Figure S2: Primers and crRNAs of HPV18 screening; Figure S3: HPV18 single-step RPA-CRISPR reaction curves; Figure S4: Simulation of temperature changes in the reaction system under a heating block at 37 °C; Figure S5: Schematic diagram of the PDMS connector coupling with the instrument during microfluidic chip insertion; Figure S6: ImageJ quantification of inter-chamber cross-contamination; Figure S7: Flow termination of a high-viscosity sample at the PTFE interface; Figure S8: Internal structure diagram of the Ccard; Figure S9: Temperature change curve of the heating block after reaction initiation; Figure S10: Ccard detection sensitivity for different FAM fluorescence concentrations; Figure S11: Ccard instrument circuit board principle diagram; Figure S12: PCB diagram of the control section of the Ccard instrument circuit board; Figure S13: PCB diagram of the optical path section of the Ccard instrument circuit board; Figure S14: Ccard software interface diagram; Figure S15: Real-time fluorescence curves of nucleic acid standards at different concentration gradients on Ccard; Figure S16: Negative control panel for the Ccard single-step RPA-CRISPR/Cas13a assay (HPV16); Figure S17: Matrix-effect evaluation of Ccard by spike-in of HPV targets into negative cervicovaginal swab lysate; Figure S18: Test results of lyophilized reagents stored at 4 °C and room temperature for 30 days; Figure S19: Real-time fluorescence curves of clinical samples on Ccard; Figure S20: Real-time fluorescence curves of clinical samples on qPCR; Table S1: The reagents used in this study; Table S2: Reagent composition of the on-chip lyophilized single-step RPA-CRISPR/Cas13a reaction; Table S3: Structural dimensions and material specifications of the Ccard microfluidic chip; Table S4: Platform comparison; Table S5: Comparison of single- and two-step CRISPR detection; Table S6: The sequences of HPV16 and HPV18 pseudovirus L1 genes; Table S7: The sequences of primers and CRISPR crRNA for HPV detection used in this study; Table S8: Liquid-volume balance of the Ccard microfluidic chip; Table S9: Material cost table for Ccard; Table S10: Comparison of existing HPV detection technologies. References [15,19,27,28,37,38,39,40,41,42,43,44,45,46,47] are cited in the Supplementary Materials.
