# Supplementary material for: Integrated Microfluidic CRISPR-Based Diagnostic Device for Rapid Testing of HPVs
Source: Biosensors (Basel). 2026 Oct 11;16(10):571. doi: 10.3390/bios16100571 (PMC13702769; doi:10.3390/bios16100571)
Supplement: Supplementary file 1 [file biosensors-16-00571-s001.zip › biosensors-4565780-supplementary.pdf]

Supporting Information

Integrated Microfluidic CRISPR-Based Diagnostic Device for Rapid Testing of HPVs

Tianci Wang [1], Lingyun Li [1], Yunxiang Wang [1], Yongjun Han [2], Teng Li [3], Chaoran Yu [3], Yufeng Lou [4], Zhen Rong [1], Kai Lin [5,*] and Shengqi Wang [1,6,*]

[1] Bioinformatics Center of AMMS, Beijing 100850, P. R. China.
[2] The Second Affiliated Hospital of Henan University of Traditional Chinese Medicine, Zhengzhou 450003, P. R. China.
[3] Department of Pathology, Air Force Medical Center, Air Force Medical University, Beijing 100142, P. R. China.
[4] Department of Laboratory Medicine, The First Affiliated Hospital, Zhejiang University School of Medicine, Hangzhou 310003, P. R. China.
[5] Department of Clinical Laboratory, Air Force Medical Center, Air Force Medical University, Beijing 100142, P. R. China.
[6] State Key Laboratory of Kidney Diseases, Beijing 100853, P. R. China.

Corresponding Author

*Shengqi Wang, E-mail: sqwang1007@163.com.

*Kai Lin, E-mail:ruth1101@163.com.

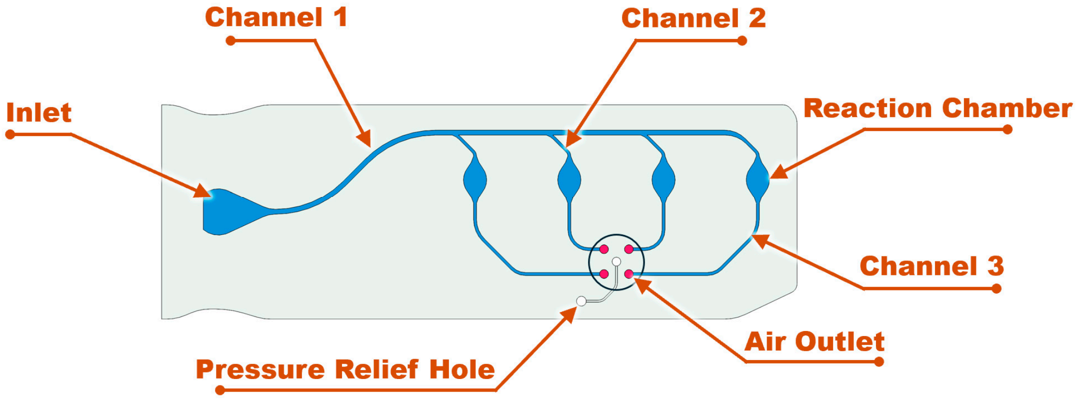

Figure S1. Schematic diagram of the Ccard microfluidic chip. Annotated layout showing the inlet reservoir, Channel 1 (main distribution channel), Channel 2 (metering branches), Channel 3 (fishbone-shaped isolation channel), four reaction chambers, air outlet, and pressure-relief holes. Structural dimensions and material specifications are listed in Supplementary Table 3.

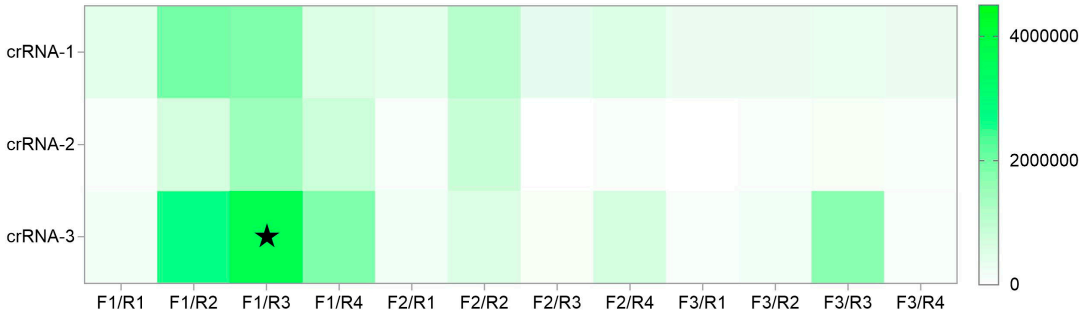

Figure S2. Primers and crRNAs of HPV18 screening. The fluorescence intensity at 30 minutes of HPV18 L1 gene was tested by plate reader using the single-step RPA-CRISPR/Cas13a reaction to screen RPA primers and Cas13a crRNA.Target: HPV18 L1 gene of 100 copies/μL.

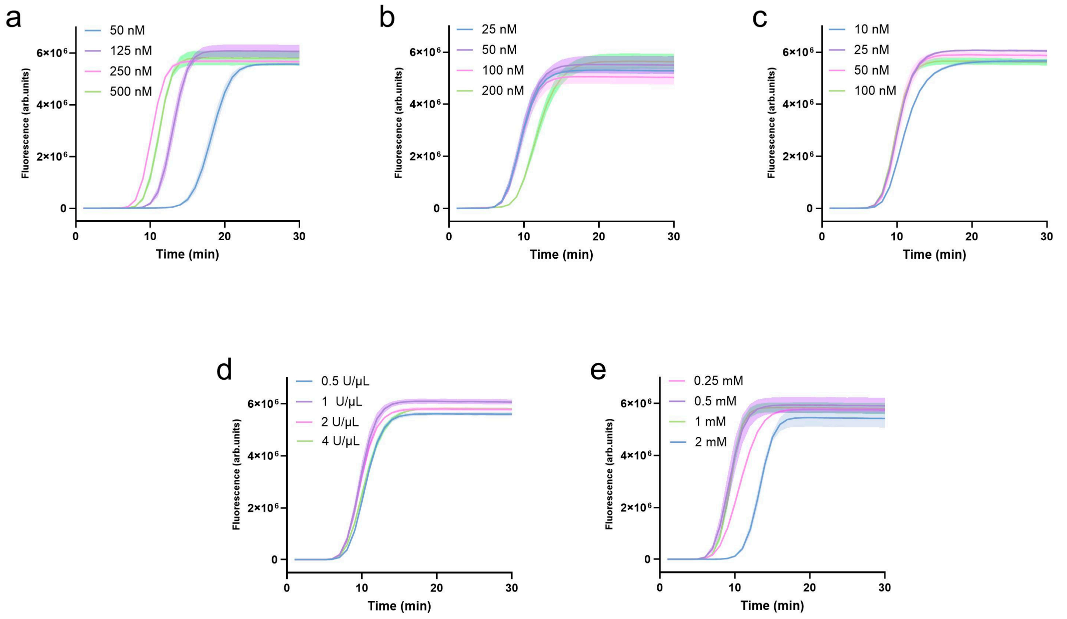

Figure S3. HPV18 single-step RPA-CRISPR reaction curve diagram (a) Primer concentrations were tested at 50, 125, 250, and 500 nM. (b) LwaCas13a concentrations were tested at 25, 50, 100, and 200 nM. (c) crRNA concentrations were tested at 10, 25, 50, and 100 nM. (d) T7 RNA polymerase concentrations were tested at 0.5, 1, 2, and 4 U/μL. (e) rNTP concentrations were tested at 0.25, 0.5, 1, and 2 mM.

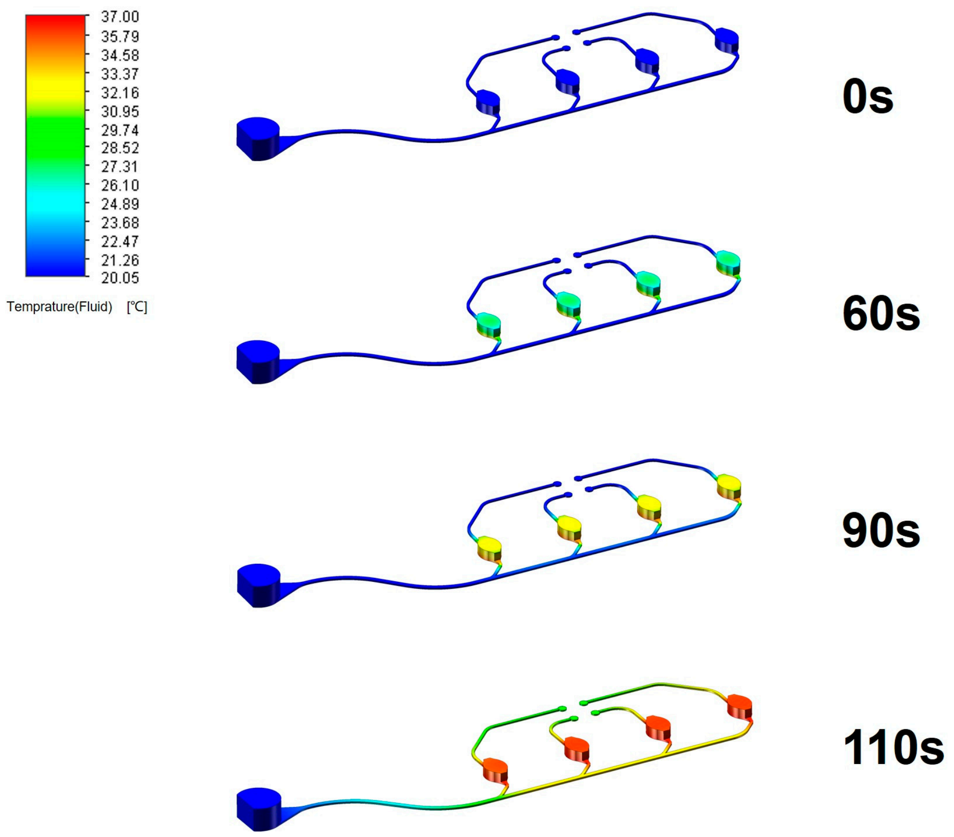

Figure S4. Simulation of temperature changes in the reaction system under a heating block at 37 °C.

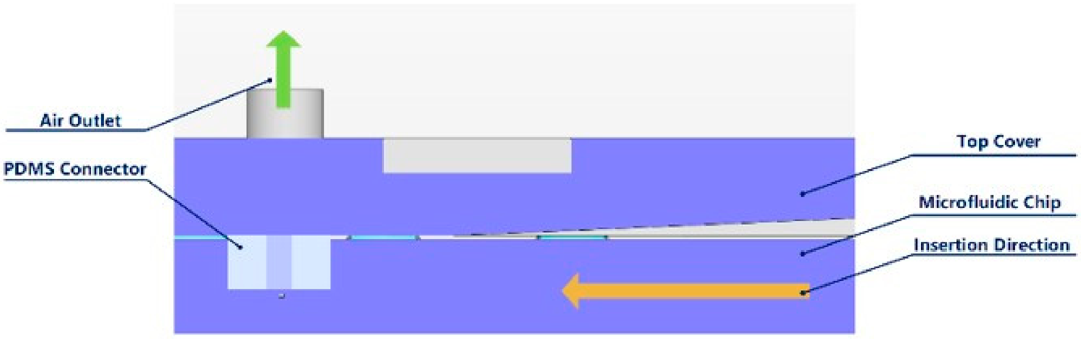

Figure S5. Schematic diagram of PDMS connector coupling with the instrument during microfluidic chip insertion.

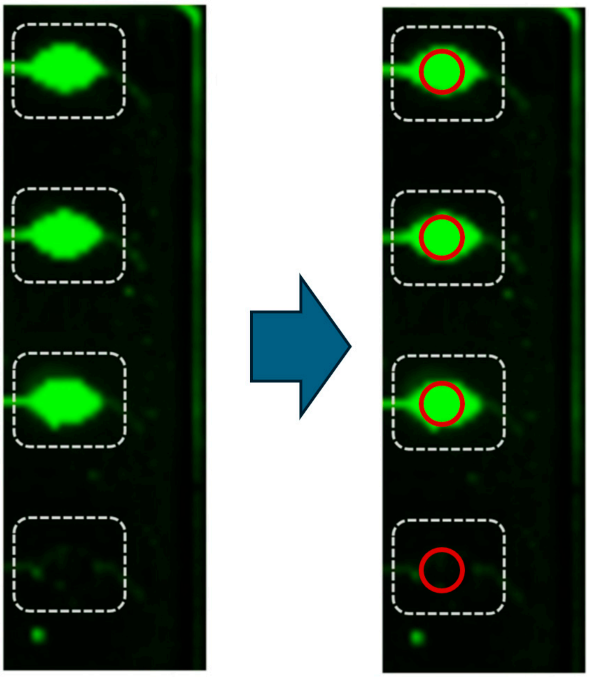

Figure S6. ImageJ quantification of inter-chamber cross-contamination.

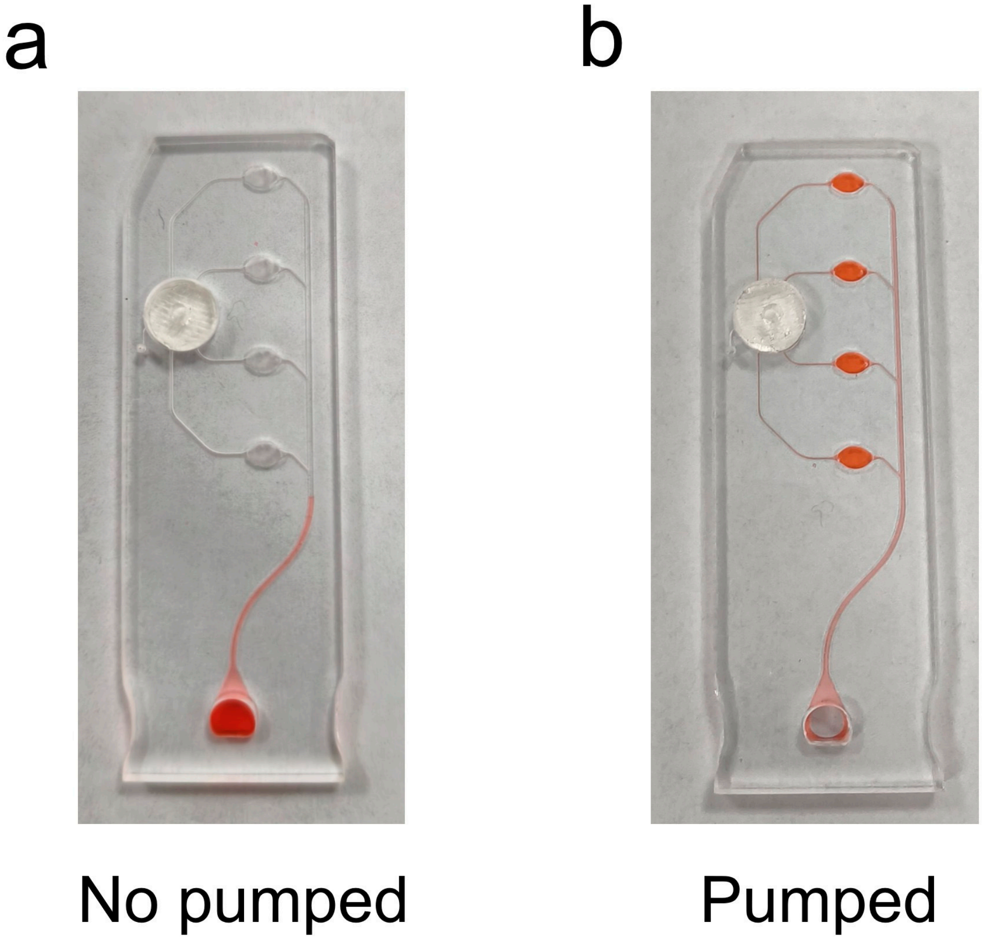

Figure S7. Flow termination of a high-viscosity sample at the PTFE interface. A 40% (v/v) glycerol solution (~4.8 mPa • s) was loaded into the chip under negative pressure. The flow stopped at the PTFE membrane without breakthrough, confirming the passive volumetric metering of the Ccard chip.

a b

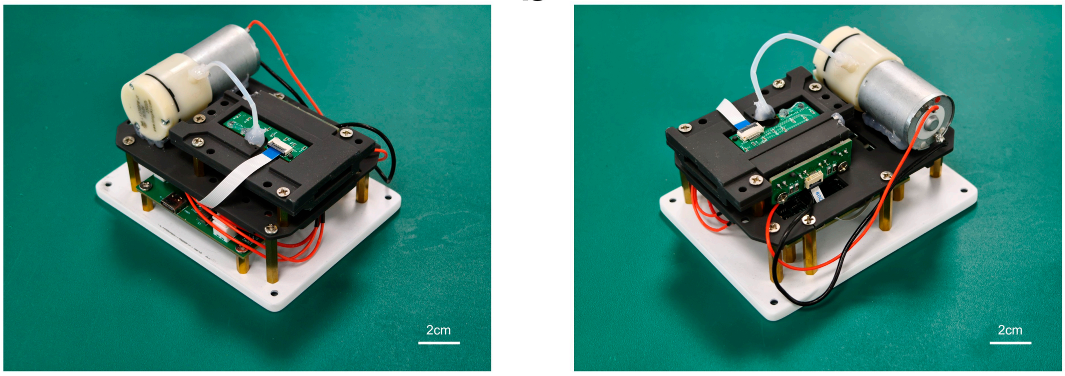

Figure S8. Internal structure diagram of the Ccard. (a) Left View (b) Right View

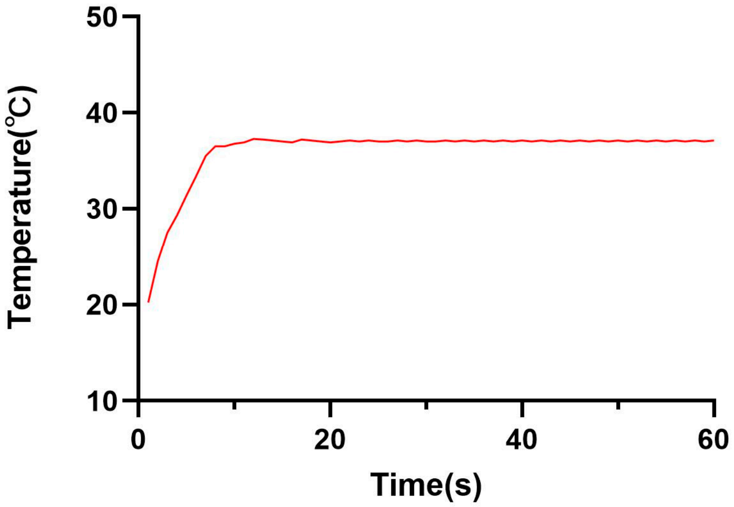

Figure S9. Temperature change curve of the heating block after reaction initiation.

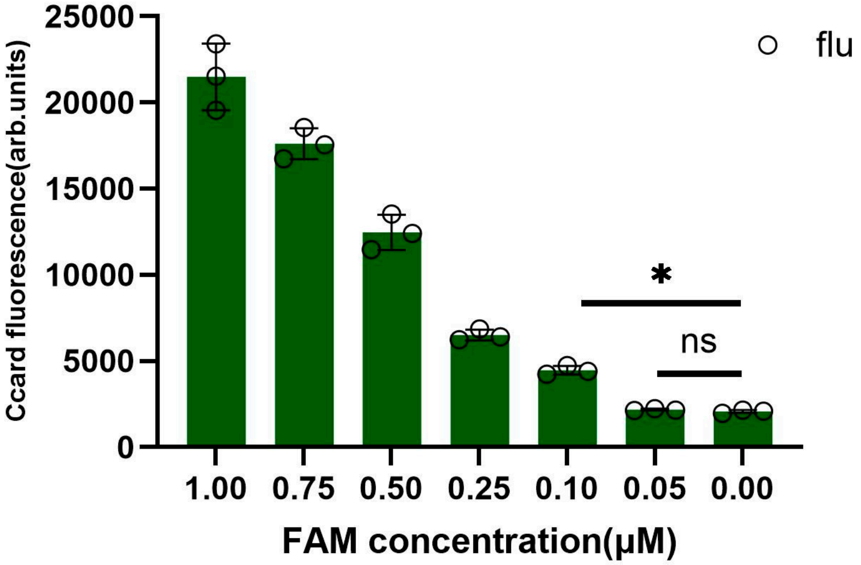

Figure S10. Ccard detection sensitivity for different FAM fluorescence concentrations.

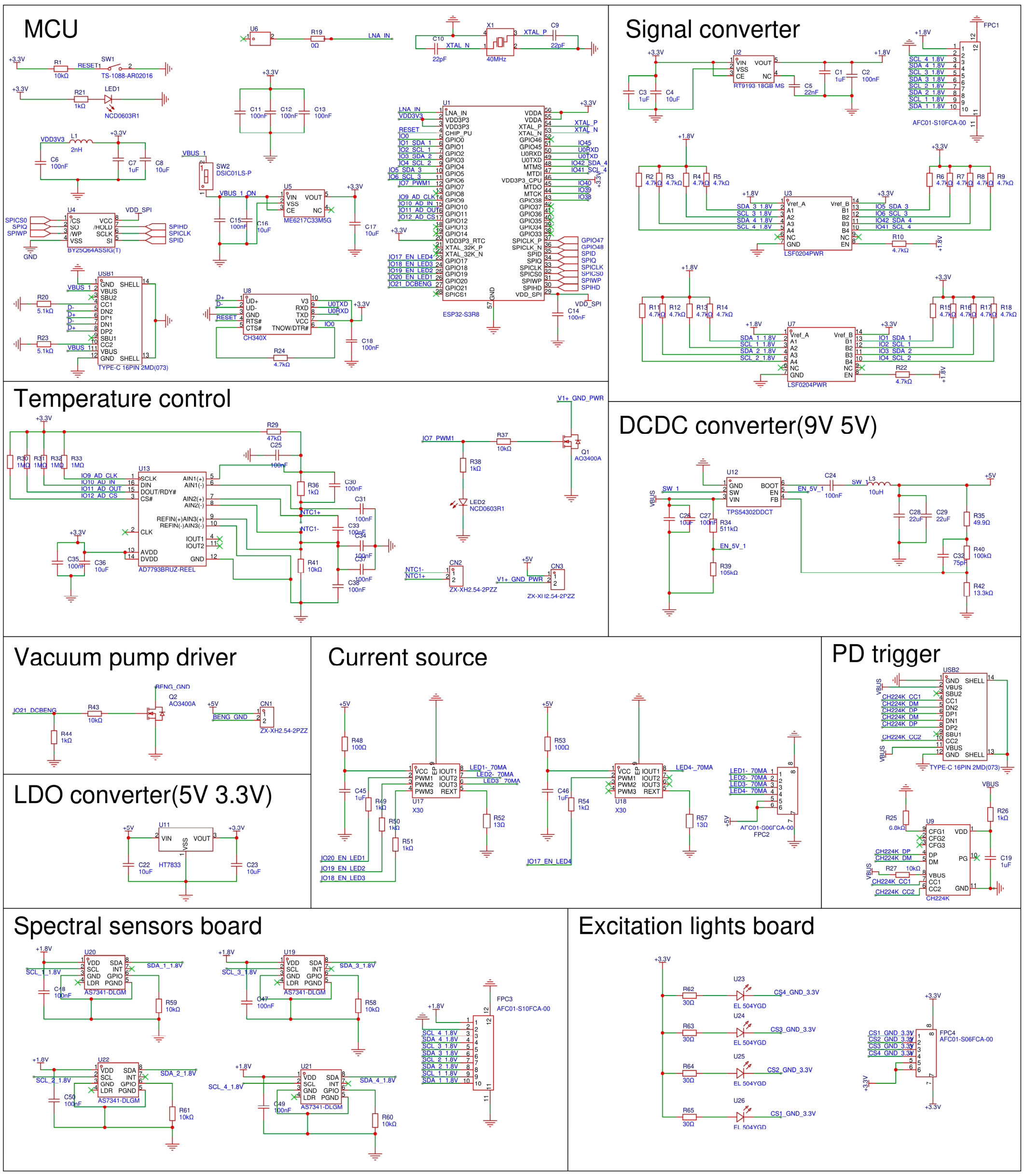

Figure S11. Ccard instrument circuit board principle diagram.

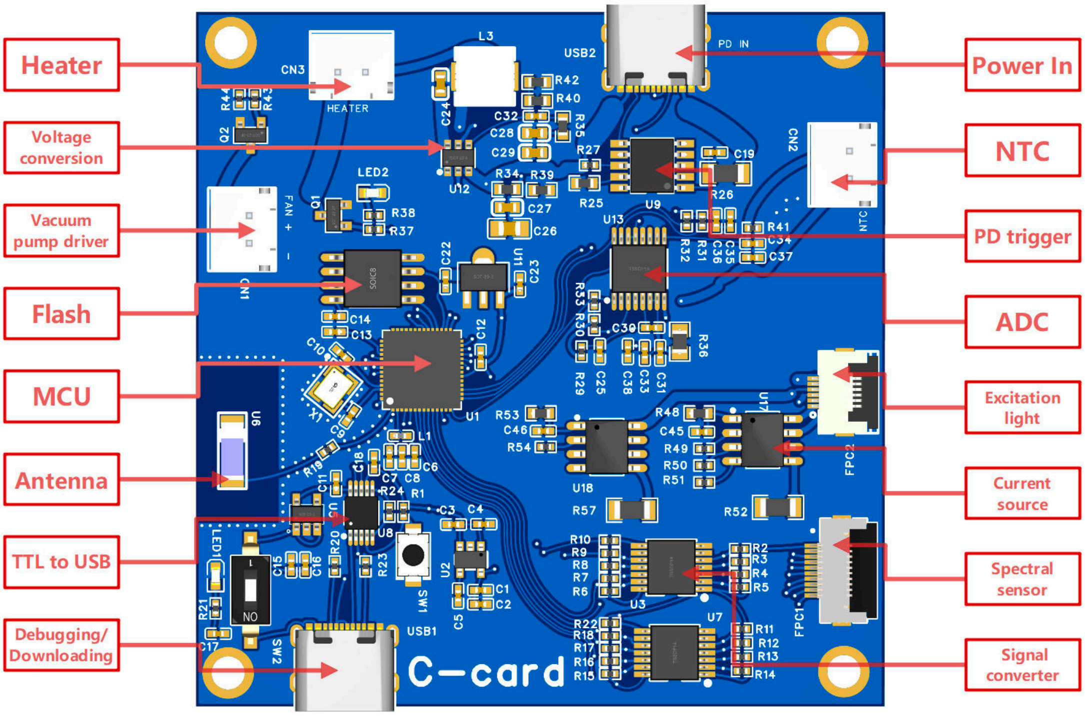

Figure S12. PCB diagram of the control section of the Ccard instrument circuit board.

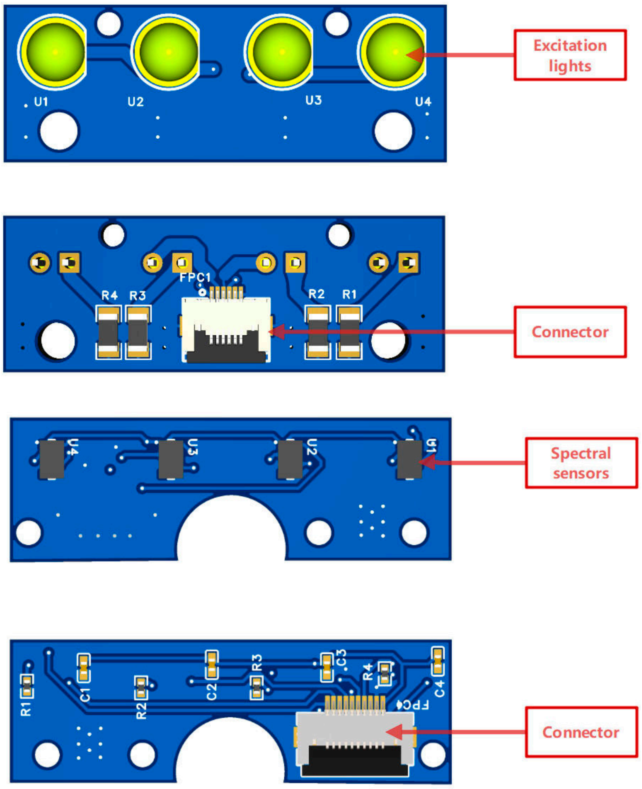

Figure S13. PCB diagram of the optical path section for the Ccard instrument circuit board. The light source excitation module is located on the upper side, and the fluorescence detection module is located on the lower side.

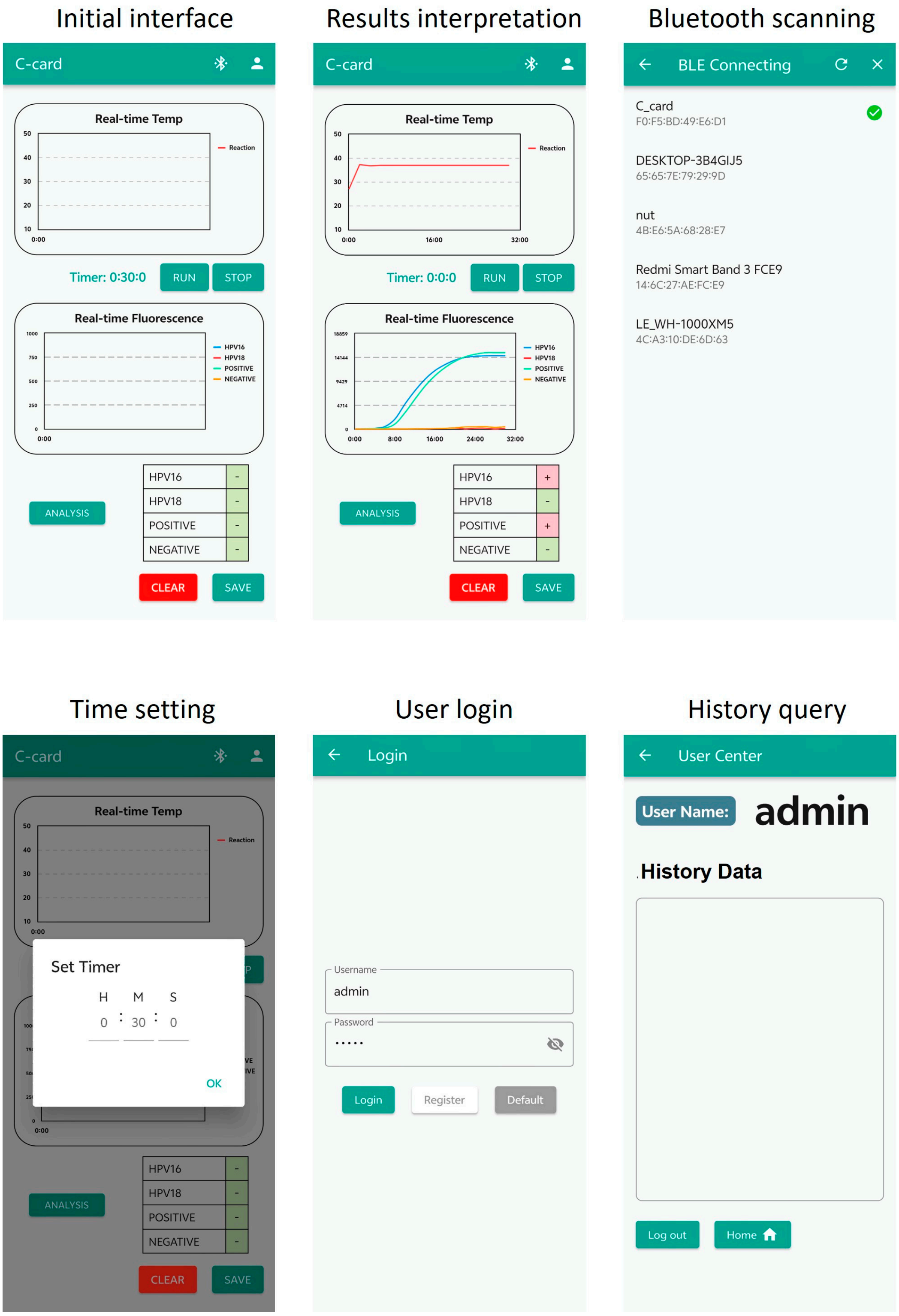

Figure S14. Ccard software interface diagram.

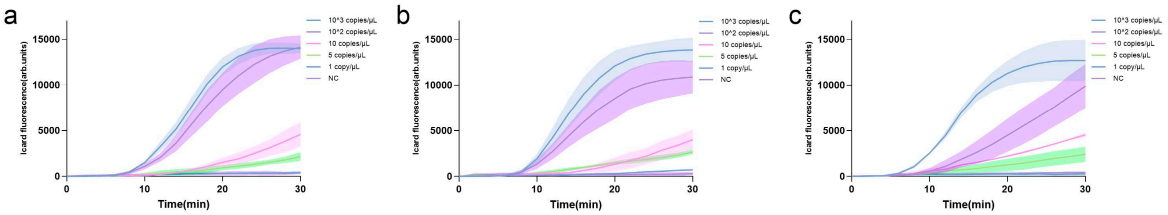

Figure S15. Real-time fluorescence curve of nucleic acid standards at different concentration gradients on Ccard. (a) Sensitivity of HPV16 Detection. (b) Sensitivity of HPV18 Detection. (c) Sensitivity of GAPDH Detection.

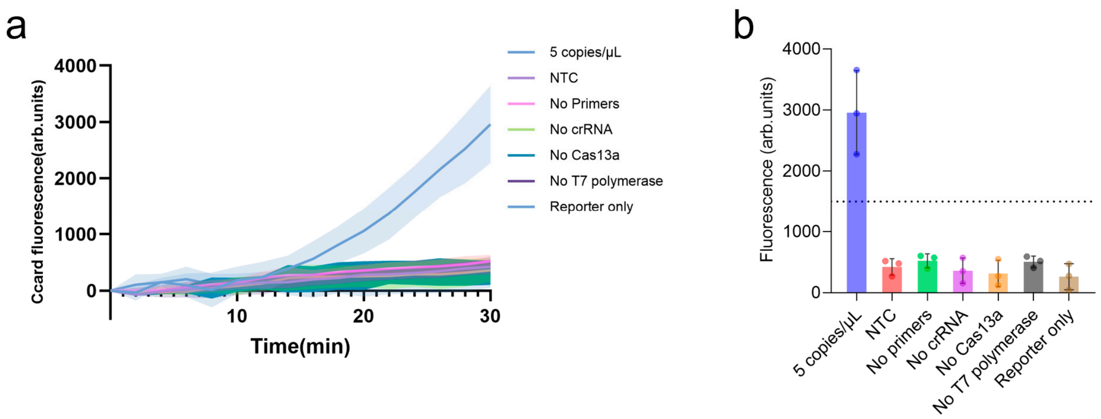

Figure S16. Negative control panel for the Ccard single-step RPA-CRISPR/Cas13a assay (HPV16). (a) Real-time fluorescence curves of the positive control (HPV16, 5 copies/μL) and six negative-control reactions: no target template (NTC, prepared in lysis buffer and serving also as the lysis-buffer blank), no primers, no crRNA, no Cas13a, no T7 RNA polymerase, and reporter only. Shaded areas represent mean ± SD (n = 3). (b) Endpoint fluorescence at 30 min for each condition. Bars represent the mean; dots represent individual replicates (n = 3). The dashed line indicates the positive threshold (cutoff = 1500).

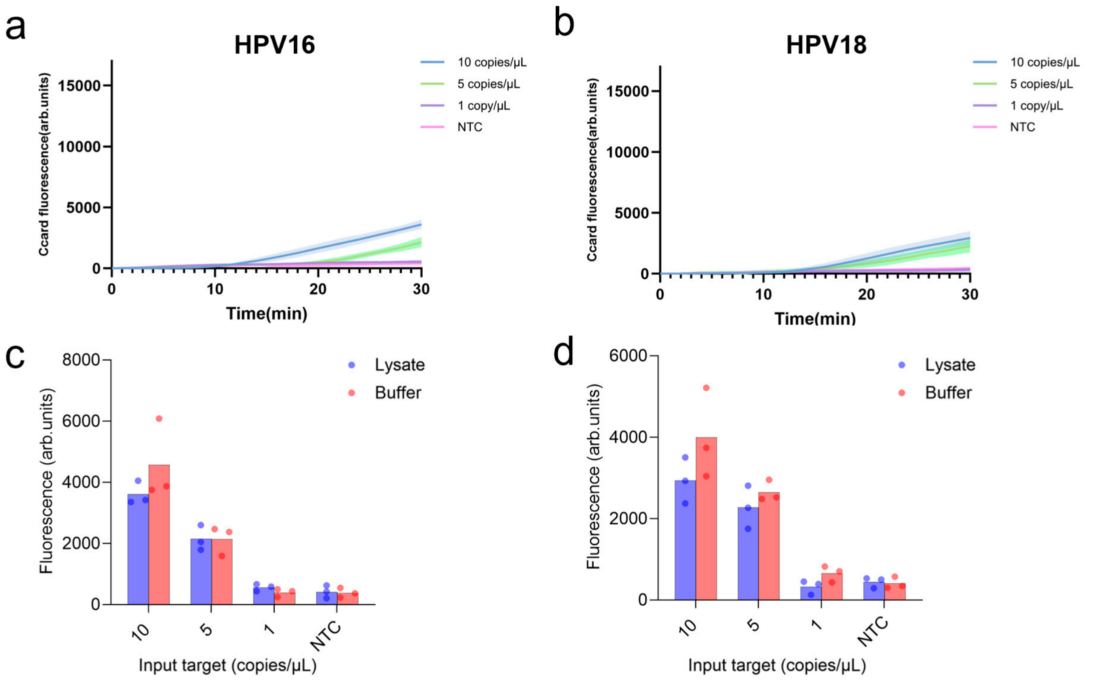

Figure S17. Matrix-effect evaluation of Ccard by spike-in of HPV targets into negative cervicovaginal swab lysate. (a-b) Real-time fluorescence curves of (a) HPV16 and (b) HPV18 targets (10, 5, 1 copies/μL and NTC) spiked into negative swab lysate. (c-d) Endpoint fluorescence (30 min) of (c) HPV16 and (d) HPV18 prepared in swab lysate versus buffer. Bars represent the mean and dots individual replicates (n = 3). NTC, no-template control.

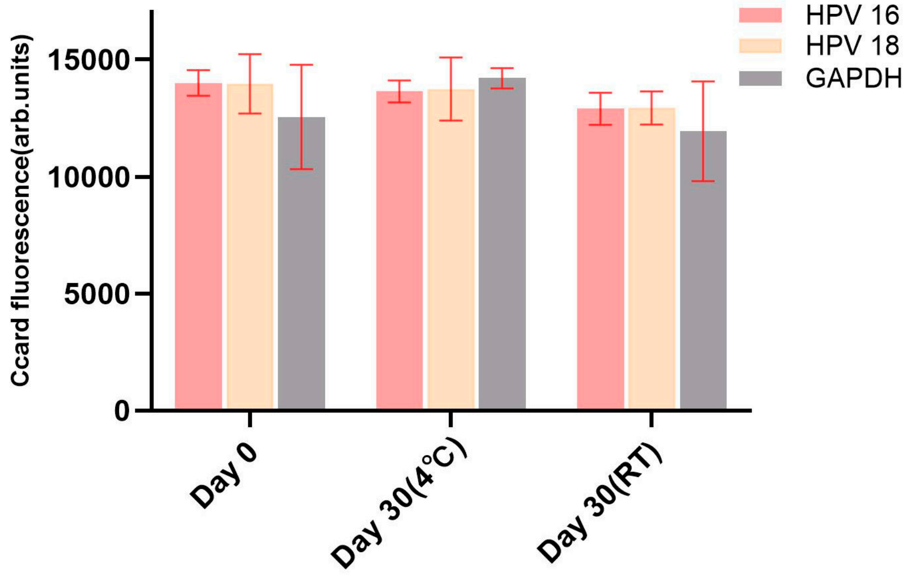

Figure S18. Test results of lyophilized reagents stored at 4 °C and room temperature for 30 days.

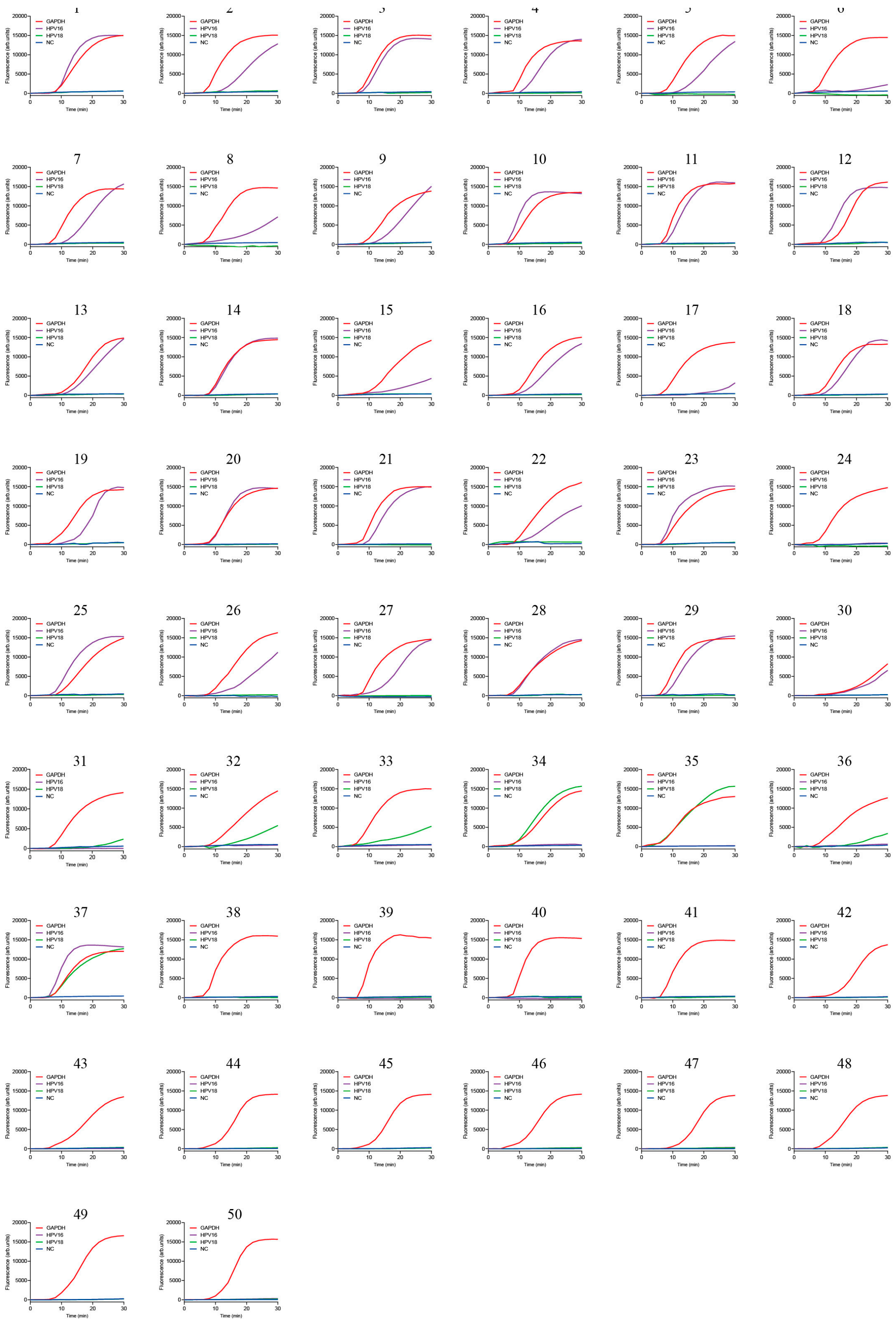

Figure S19. Real-time fluorescence curve of clinical samples on Ccard.

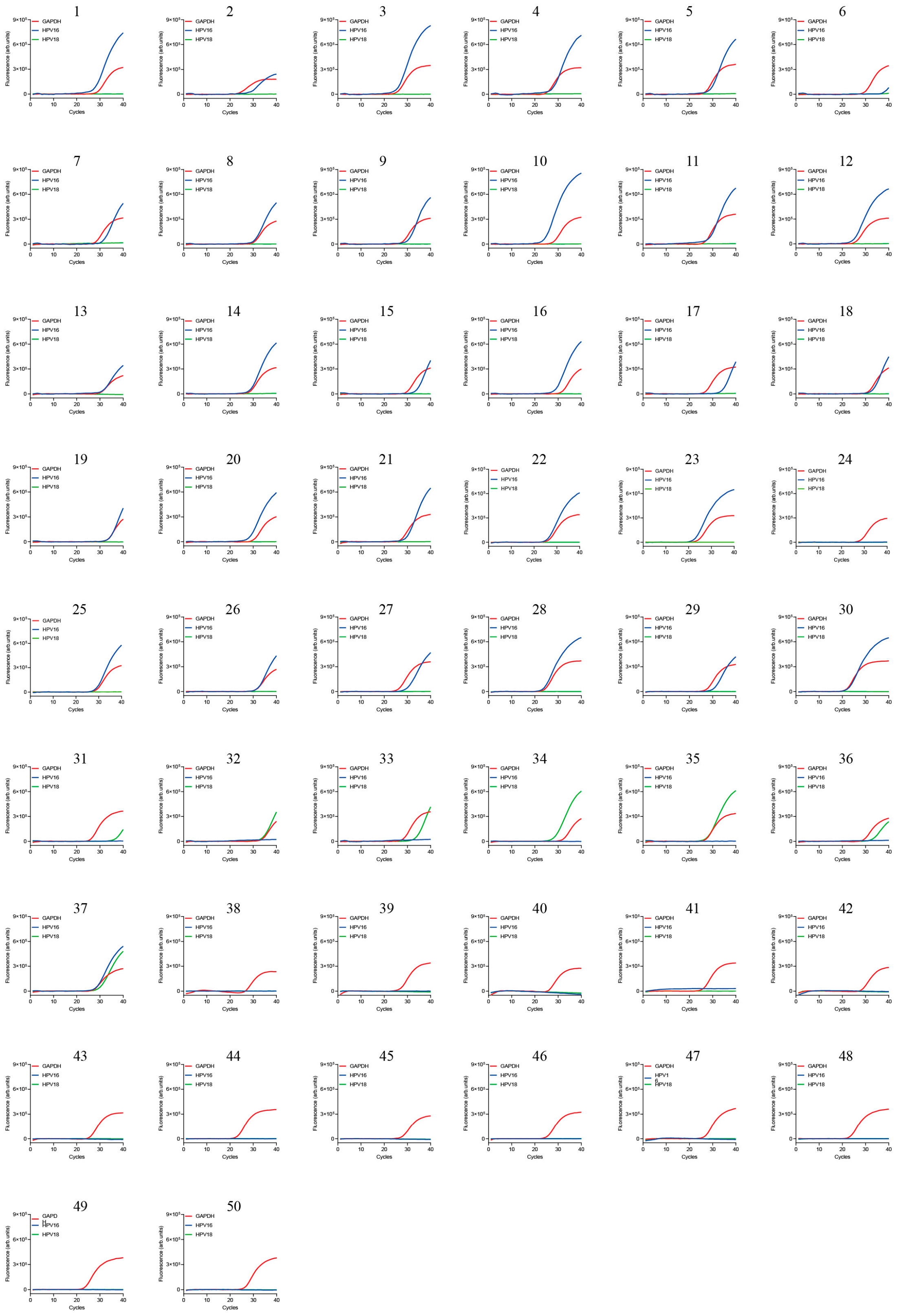

Figure S20. Real-time fluorescence curve of clinical samples on qPCR.

Supplementary Table S1. The reagents used in this study.

Reagent	Reaction	Manufacturer	Cat.no.	Stock Concentration
RPA lyophilized pellets	Single-step RPA-Cas13 assay	TwistDx™	TABAS03KIT	/
Rehydration buffer	Single-step RPA-Cas13 assay	TwistDx™	TABAS03KIT	/
RNase Inhibitor	Single-step RPA-Cas13 assay	Solarbio® Life Science	R8061	40 U/µL
rNTP mix	Single-step RPA-Cas13 assay	New England Biolabs® Inc.	N0466	25 mM
T7 RNA polymerase	Single-step RPA-Cas13 assay	New England Biolabs® Inc.	M0251	50 U/µL
RNase H	Single-step RPA-Cas13 assay	New England Biolabs® Inc.	M0297	5 U/µL
LwaCas13a	Single-step RPA-Cas13 assay	Tolo Biotech Co., Ltd	32117-01	10 µM
HPV16 pseudovirus	Single-step RPA-Cas13 assay	Fubio	FNDV4994	/
HPV18 pseudovirus	Single-step RPA-Cas13 assay	Fabio	FNDV4995	/
Human Genomic DNA Quantitative Standard	Single-step RPA-Cas13 assay	BNCC	BNCC372385	
DNase/RNase-free water	/	Solarbio® Life Science	R1600	/
HiScribe ™ T7 High Yield RNA Synthesis Kit	IVT	New England Biolabs® Inc.	E2040	/
Standard Taq buffer	IVT	New England Biolabs® Inc.	B9014	10×
RNA Clean & Concentrator-5 Kit	IVT	ZYMO RESEARCH	R1013	/

RQ1 RNase-Free DNase	IVT	Promega	M6101	10 U/μL
Qubit™ RNA Broad Range Assay Kits	IVT	Thermo Fisher Scientfic™	Q10211	/
TIANamp Virus DNA/RNA kit	Viral extraction	Tiangen Biotech	DP315	/
Sample Release Agent	Viral lysis	GenDx Biotech	GD005	/
D-(+)-Trehalose Anhydrous	Lyophilization	Mackin	D807342-100g	/
Mannitol	Lyophilization	Aladdin	M108829-500g	/
Poly(ethylene glycol)	Lyophilization	Sigma-Aldrich	81300-1KG	/
Premix Ex Taq (Probe qPCR)	qPCR	TaKaRa	RR390	/
TIANamp Genomic DNA kit	Sample extraction	Tiangen Biotech	DP304	/

Supplementary Table S2. Reagent composition of the on-chip lyophilized single-step RPA-CRISPR/Cas13a reaction.

**Reagent**	**Stock concentration**	**Volume added (per 30 µL pre-mix)**	**Amount lyophilized per chamber**	**Final concentration (8 µL reaction)**
RPA lyophilized powder	Commercial (TwistDx, TABAS03KIT)	1 tube, resuspended in the 30 µL pre-mix	0.07 tube (= 2.1/30)	Per manufacturer's instructions
RNase inhibitor	40 U/µL	2.86 µL	8 U	1 U/µL
rNTP mix	25 mM	2.29 µL	4 nmol	0.5 mM
T7 RNA polymerase	50 U/µL	2.29 µL	8 U	1 U/µL
Forward primer	10 µM	2.86 µL	2 pmol	250 nM
Reverse primer	10 µM	2.86 µL	2 pmol	250 nM
crRNA	10 µM	0.29 µL	0.2 pmol	25 nM
Reporter FAM-UUUUU-BHQ1	100 µM	1.14 µL	8 pmol	1 µM
LwaCas13a	10 µM	0.57 µL	0.4 pmol	50 nM
Lyoprotectant (5×)	5×	6.00 µL	—	— (1× in the pre-mix)
Nuclease-free water	—	To 30 µL	—	—

Supplementary Table S3. Structural dimensions and material specifications of the Ccard microfluidic chip.

Feature	Dimension
Channel 1	0.5 mm wide × 0.2 mm high
Channel 2	0.3 mm wide × 0.2 mm high, 3 mm long
Channel 3	0.3 mm wide × 0.2 mm high; long side 18 mm, short side 8 mm
Reaction chamber	1.2 mm deep, 8 μL
Inlet reservoir	48 μL
Outlet	7.5 mm outer diameter, containing four holes of 0.9 mm diameter
Pressure-relief vent	Two holes of 0.9 mm diameter
PTFE membrane	7.4 mm cut diameter; 0.2 mm (200 ± 60 μm) thick; 0.1 μm pore size (Cobetter MFPT-1047, hydrophobic); average water breakthrough pressure ≥450 kPa
PSA tape	PP, 0.1 mm thick (Servicebio G6065-25)

Supplementary Table S4. Platform Comparison.

Platform Name	Assay Mode	Driving Mechanism	Fluidic Control Strategy	Portability	Reference
**V-PAM**	Biofluid Filling	Negative Pressure	PDMS Gas permeation	Module/Integrated	[1]
**IoT-based POC Device**	RT-LAMP	Negative/Positive Pressure (Syringe)	Wax valves / Aliquoting	320 g	[2]
**Lift-CM**	RPA/LAMP + CRISPR-Cas12a	Centrifugal Force	Integrated Lift-heater mechanism	3.15 kg (Integrated)	[3]
**ORCMB**	RPA-CRISPR/Cas12a	Centrifugal Force	Sacrificial chambers / Capillary channels	2.88 kg (Portable)	[4]
**CARE**	RPA-CRISPR/Cas12a	Pressure (Stepper motor)	Hermetically sealed microfluidic chip	Portable instrument	[5]
**PLACID**	LAMP-CRISPR/Cas12a	Capillary Action	Wax barriers (Paper-based)	Ultra-light	[6]
**Ccard**	**Single-step RPA-Cas13a**	**Negative Pressure**	**PTFE Blocking**	**320 g (Handheld)**	**This Work (Ccard)**

Supplementary Table S5. Comparison of Single- and Two-Step CRISPR Detection.

Platform Name	Target	Sample Process	Assay Mode	Readout	LOD	TAT (min)	Contamination Risk	Reference
**SHERLOCK**	Zika / Ebola	Standard extraction	Traditional two-step RT-RPA + Cas13a	Fluorescence / Lateral flow	~1 copy/µL	> 60	High (Open-tube)	[7]
**DETECTR**	HPV (DNA)	Standard extraction	Traditional two-step RPA + Cas12a	Fluorescence	~10 copies/µL	> 60	High (Open-tube)	[8]
**RC-LFA**	HPV16/18	Crude lysate	Sequential two-step RPA + Cas12a/13a	Lateral flow assay (LFA)	10 copies/µL	~50	High (LFA exposure)	[9]
**CRISPRD**	HPV16/18	Extraction (LAMP-based)	Multiplexed one-pot Cas12b/13a	Real-time fluorescence	10 copies/µL	60	Negligible (One-pot)	[10]
**One-tube RPA**	Monkeypox	Extraction / Swab lysate	One-pot/Two-step RPA + Cas12a	Real-time fluorescence	1-10 copies/rxn	~45	Low (Physical separation)	[11]
**Ccard**	**HPV16/18, GAPDH**	**Direct lysis (Extraction-free)**	**Integrated single-step RPA-Cas13a**	**Real-time fluorescence (IoT)**	**5 copies/µL**	**~30**	**Negligible (Closed)**	**This Study**

Supplementary Table S6. The sequences of HPV16 and HPV18 pseudovirus L1 genes.

Virus	Sequence (5’-3’)
HPV16	ATGTCTCTTTGGCTGCCTAGTGAGGCCACTGTCTACTTGCCTCCTGTCCCAGT<br>ATCTAAGGTTGTAAGCACGGATGAATATGTTGCACGCACAAACATATATTAT<br>CATGCAGGAACATCCAGACTACTTGCAGTTGGACATCCCTATTTTCCTATTA<br>AAAAACCTAACAATAACAAAATATTAGTTCCTAAAGTATCAGGATTACAAT<br>ACAGGGTATTTAGAATACATTTACCTGACCCCAATAAGTTTGGTTTTCCTGA<br>CACCTCATTTTATAATCCAGATACACAGCGGCTGGTTTGGGCCTGTGTAGGT<br>GTTGAGGTAGGTCGTGGTCAGCCATTAGGTGTGGGCATTAGTGGCCATCCTT<br>TATTAAATAAATTGGATGACACAGAAAATGCTAGTGCTTATGCAGCAAATG<br>CAGGTGTGGATAATAGAGAATGTATATCTATGGATTACAAACAAACACAAT<br>TGTGTTTAATTGGTTGCAAACCACCTATAGGGGAACACTGGGGCAAAGGAT<br>CCCCATGTACCAATGTTGCAGTAAATCCAGGTGATTGTCCACCATTAGAGTT<br>AATAAACACAGTTATTCAGGATGGTGATATGGTTGATACTGGCTTTGGTGCT<br>ATGGACTTTACTACATTACAGGCTAACAAAAGTGAAGTTCCACTGGATATTT<br>GTACATCTATTTGCAAATATCCAGATTATATTAAAATGGTGTCAGAACCATA<br>TGGCGACAGCTTATTTTTTTATTTACGAAGGGAACAAATGTTTGTTAGACAT<br>TTATTTAATAGGGCTGGTGCTGTTGGTGAAAATGTACCAGACGATTTATACA<br>TTAAAGGCTCTGGGTCTACTGCAAATTTAGCCAGTTCAAATTATTTTCCTAC<br>ACCTAGTGGTTCTATGGTTACCTCTGATGCCCAAATATTCAATAAACCTTAT<br>TGGTTACAACGAGCACAGGGCCACAATAATGGCATTTGTTGGGGTAACCAA<br>CTATTTGTTACTGTTGTTGATACTACACGCAGTACAAATATGTCATTATGTGC<br>TGCCATATCTACTTCAGAAACTACATATAAAAATACTAACTTTAAGGAGTAC<br>CTACGACATGGGGAGGAATATGATTTACAGTTTATTTTTCAACTGTGCAAAA<br>TAACCTTAACTGCAGACGTTATGACATACATACATTCTATGAATTCCACTAT<br>TTTGGAGGACTGGAATTTTGGTCTACAACCTCCCCCAGGAGGCACACTAGA<br>AGATACTTATAGGTTTGTAACATCCCAGGCAATTGCTTGTCAAAAACATACA<br>CCTCCAGCACCTAAAGAAGATCCCCTTAAAAAATACACTTTTTGGGAAGTA<br>AATTTAAAGGAAAAGTTTTCTGCAGACCTAGATCAGTTTCCTTTAGGACGCA<br>AATTTTTACTACAAGCAGGATTGAAGGCCAAACCAAAATTTACATTAGGAA<br>AACGAAAAGCTACACCCACCACCTCATCTACCTCTACAACTGCTAAACGCA<br>AAAAACGTA
HPV18	ATGTGCCTGTATACACGGGTCCTGATATTACATTACCATCTACTACCTCTGT<br>ATGGCCCATTGTATCACCCACAGCCCCTGCCTCTACACAGTATATTGGTATA<br>CATGGTACACATTATTATTTGTGGCCATTATATTATTTTATTCCTAAGAAACG<br>TAAACGTGTTCCCTATTTTTTTGCAGATGGCTTTGTGGCGGCCTAGTGACAA<br>TACCGTATATCTTCCACCTCCTTCTGTGGCAAGAGTTGTAAATACCGATGAT<br>TATGTGACTCGCACAAGCATATTTTATCATGCTGGCAGCTCTAGATTATTAA<br>CTGTTGGTAATCCATATTTTAGGGTTCCTGCAGGTGGTGGCAATAAGCAGGA<br>TATTCCTAAGGTTTCTGCATACCAATATAGAGTATTTAGGGTGCAGTTACCT<br>GACCCAAATAAATTTGGTTTACCTGATACTAGTATTTATAATCCTGAAACAC<br>AACGTTTAGTGTGGGCCTGTGTTGGAGTGGAAATTGGCCGTGGTCAGCCTTT

	AGGTGTTGGCCTTAGTGGGCATCCATTTTATAATAAATTAGATGACACTGAA<br>AGTTCCCATGCCGCCACGTCTAATGTTTCTGAGGACGTTAGGGACAATGTGT<br>CTGTAGATTATAAGCAGACACAGTTATGTATTTTGGGCTGTGCCCCTGCTAT<br>TGGGGAACACTGGGCTAAAGGCACTGCTTGTAAATCGCGTCCTTTATCACAG<br>GGCGATTGCCCCCCTTTAGAACTTAAAAACACAGTTTTGGAAGATGGTGATA<br>TGGTAGATACTGGATATGGTGCCATGGACTTTAGTACATTGCAAGATACTAA<br>ATGTGAGGTACCATTGGATATTTGTCAGTCTATTTGTAAATATCCTGATTATT<br>TACAAATGTCTGCAGATCCTTATGGGGATTCCATGTTTTTTTGCTTACGGCGT<br>GAGCAGCTTTTTGCTAGGCATTTTTGGAATAGAGCAGGTACTATGGGTGACA<br>CTGTGCCTCAATCCTTATATATTAAAGGCACAGGTATGCGTGCTTCACCTGG<br>CAGCTGTGTGTATTCTCCCTCTCCAAGTGGCTCTATTGTTACCTCTGACTCCC<br>AGTTGTTTAATAAACCATATTGGTTACATAAGGCACAGGGTCATAACAATG<br>GTGTTTGCTGGCATAATCAATTATTTGTTACTGTGGTAGATACCACTCGCAG<br>TACCAATTTAACAATATGTGCTTCTACACAGTCTCCTGTACCTGGGCAATAT<br>GATGCTACCAAATTTAAGCAGTATAGCAGACATGTTGAGGAATATGATTTG<br>CAGTTTATTTTTCAGTTGTGTACTATTACTTTAACTGCAGATGTTATGTCCTA<br>TATTCATAGTATGAATAGCAGTATTTTAGAGGATTGGAACTTTGGTGTTCCC<br>CCCCCGCCAACTACTAGTTTGGTGGATACATATCGTTTTGTACAATCTGTTG<br>CTATTACCTGTCAAAAGGATGCTGCACCGGCTGAAAATAAGGATCCCTATG<br>ATAAGTTAAAGTTTTGGAATGTGGATTTAAAGGAAAAGTTTTCTTTAGACTT<br>AGATCAATATCCCCTTGGACGTAAATTTTTGGTTCAGGCTGGATTGCGTCGC<br>AAGCCCACCATAGGCCCTCGCAAACGTTCTGCTCCATCTGCCACTACGTCTT<br>CTAAACCTGCCAAGCGTGTGCGTGTACGTGCCAGGAAGTAA

Supplementary Table S7. The sequences of primers and CRISPR crRNA for HPV detection used in this study.

Name	Sequence (5'- 3')
**HPV16-RPA-F**	*cctctaatacgactcactataggg*CTGTCCCAGTATCTAAGGTTGTAAGCACGG
**HPV16-RPA-R**	GTATCTGGATTATAAAATGAGGTGTCAGGA
**HPV16-crRNA**	GAUUUAGACUACCCCAAAAACGAAGGGGACUAAAACUAGGAAAAUAGGGAUGUCCAACUGCAAG
**HPV18-RPA-F1**	CCTCTAATACGACTCACTATAGGGCCCTGCTATTGGGGAACACTGGGCTAAAG
**HPV18-RPA-F2**	CCTCTAATACGACTCACTATAGGGCTATTGGGGAACACTGGGCTAAAGGCACTG
**HPV18-RPA-F3**	CCTCTAATACGACTCACTATAGGGACACTGGGCTAAAGGCACTGCTTGTAAATC
**HPV18-RPA-R1**	AATAGACTGACAAATATCCAATGGTACCTC
**HPV18-RPA-R2**	CTACCATATCACCATCTTCCAAAACTGTGT
**HPV18-RPA-R3**	ATCCAGTATCTACCATATCACCATCTTCCA
**HPV18-RPA-R4**	GGCACCATATCCAGTATCTACCATATCACC
**HPV18-crRNA1**	GAUUUAGACUACCCCAAAAACGAAGGGGACUAAAACAGGGGGGCAAUCGCCCUGUGAUAAAGGA
**HPV18-crRNA2**	GAUUUAGACUACCCCAAAAACGAAGGGGACUAAAACUAAAGGGGGGCAAUCGCCCUGUGAUAAA
**HPV18-crRNA3**	GAUUUAGACUACCCCAAAAACGAAGGGGACUAAAACAAGUUCUAAAGGGGGGCAAUCGCCCUGU
**GAPDH-RPA-F**	*cctctaatacgactcactataggg*TCACCAGGGCTGCTTTTAACTCTGGTAAAG
**GAPDH-RPA-R**	CATGGGTGGAATCATATTGGAACATGTAAA
**GAPDH-crRNA**	GAUUUAGACUACCCCAAAAACGAAGGGGACUAAAACAAUGAAGGGGUCAUUGAUGGCAACAAUA
**HPV16-qPCR-F**	ATACATTTACCTGACCCCAA
**HPV16-qPCR-R**	ACCTCAACACCTACACAGGC
**HPV16-qPCR-P**	TTTGGTTTTCCTGACACCTCATT
**HPV18-qPCR-F**	GTGTGTATTCTCCCTCTCCA
**HPV18-qPCR-R**	GACCCTGTGCCTTATGTAAC
**HPV18-qPCR-P**	TGGCTCTATTGTTACCTCTGACTCC
**GAPDH-qPCR-F**	ACCAGGGCTGCTTTTAACTC
**GAPDH-qPCR-R**	AACATGTAAACCATGTAGTTGAGG
**GAPDH-qPCR-P**	TATTGTTGCCATCAATGACCCCTTC
**T7-3G-IVT primer**	GAAATTAATACGACTCACTATAGGG

Supplementary Table S8. Liquid-volume balance of the Ccard microfluidic chip.

**Component**	**Volume**	**Note**
Reaction chamber (per well)	8 μL	4 chambers, 32 μL in total
Channel upstream of chambers	7.2 μL	Inlet-to-chamber microchannel
Channel downstream of chambers	3.16 μL	Chamber-to-vent/outlet microchannel
Inlet reservoir	48 μL	Sample port capacity
Outlet / dead volume	1.27 μL	4 outlet holes, total
Chamber-filling volume	≈ 35.16 μL	4 × 8 μL (chambers) + 3.16 μL (downstream channel)
Loaded volume	40 μL	23.6 μL rehydration buffer + 2 μL Mg acetate + 14.4 μL sample
Residual volume after loading	Not directly measurable	Retained in the upstream channel, which has a capacity of 7.2 μL

Supplementary Table S9. Material cost table for Ccard.

	Item	Catalog	Manufacturer	Quantity	Price($)	Cost($)
Electronical components	Analog to Digital Converter	AD7793BRUZ-REEL	ADI	1	1.944	1.944
	FPC connector	AFC01-S06FCA-00	JS	1	0.075	0.075
		AFC01-S10FCA-00		1	0.077	0.077
	Field effect transistor	AO3400A	AOS	2	0.071	0.142
	Optical sensor	AS7341-DLGM	AMS	4	4.069	16.276
	NOR FLASH	BY25Q64ASSIG(T)	BOYAMICRO	1	0.359	0.359
	Fast charging chip	CH224K	WCH	1	0.575	0.575
	USB conversion chip	CH340X		1	0.47	0.47
	Button	DSIC01LS-P	XKB Connectivity	1	0.38	0.38
	MCU	ESP32-S3R8	ESPRESSIF	1	1.887	1.887
	Linear Voltage Regulators	HT7833	HXY MOSFET	2	0.134	0.268
	Level Converter	LSF0204PWR	TI	1	0.675	0.675
	Light Emitting Diodes	NCD0603R1	MEIHU	2	0.008	0.017
	antenna	RFANT5220110A0T	Walsin	1	0.069	0.069
	DC-to-DC Converter	TPS54302DDCT	TI	1	0.218	0.218
	Button	TS-1088-AR02016	XUNPU	1	0.056	0.056
	USB connector	TYPE-C 16PIN 2MD(073)	SHOU HAN	2	0.052	0.104
	LED driver	X30	Hichips	2	0.296	0.592
	Wire-to-board headers	ZX-XH2.54-2PZZ	Megastar	3	0.024	0.072
	Passive crystal oscillator	40MHz	YXC	1	0.077	0.077
	Power inductor	10uH	cjiang	1	0.094	0.094
		2nH		1	0.008	0.008
	470 nm LED	C503B-BAN-CZ0A0452	Cree LED	4	0.689	2.756
	470 nm optical filter	/	Beijing YONGXING SENSING	1	0.193	0.193
	Temperature transducer	Mf54k	NanJing Sino Chip	1	0.055	0.055
	Resistance	0Ω	UNI-ROYAL	1	0	0
		1kΩ		8	0	0.004
		1MΩ		4	0.001	0.002
		4.7kΩ		19	0	0.009
		5.1kΩ		2	0	0.001
		6.8kΩ		1	0.001	0.001
		10kΩ		10	0	0.005
		13Ω		2	0.002	0.005
		13.3kΩ		1	0.001	0.001
		30Ω		4	0.002	0.009
		47kΩ		1	0	0
		49.9Ω		1	0.001	0.001
		100kΩ		1	0.001	0.001
		100Ω		1	0.001	0.001
		105kΩ		1	0.001	0.001
		511kΩ		1	0.001	0.001
	Capacitance	1uF	YAGEO	6	0.002	0.012
		10uF		7	0.005	0.038
		22nF		1	0.002	0.002
		22pF		2	0.001	0.002
		22uF		2	0.035	0.07
		75pF		1	0.001	0.001
		100nF		22	0.001	0.018
	Mainboard	/	Shenzhen JLC	1	0.083	0.083
	Welding	/	/	1	0.078	0.078
Mechanical componets	Shell	/	3D-print	1	2.113	2.113
	Metal components	/	Custom-made	1	2.817	2.817
	Screw	/	/	8	0.005	0.037
Microfluidic Chip	Microfluidic Chip	/	Custom-made	1	0.282	0.282
**Total**						33.034

Supplementary Table S10. Comparison of existing HPV detection technologies.

Assay	Detection Method	Detection Unit	Targets	Detection Time	LOD	Additional Equipment	Reference
CRISPRD	LAMP + CRISPR-Cas12b/13a, fluorescence	Single tube (One-pot)	16, 18, RNase P	< 1 h	10 copies/reaction	Yes	[10]
Dual-channel system	Multiplex RAA + CRISPR-Cas13a/12a, fluorescence	Tube / Portable fluorometer	16, 18	~1 h	1 copy/μL	Yes	[12]
HPV POC RPA	RPA + Lateral flow assay (LFA), colorimetry	Single tube + LFA cassette	14 hr-HPV	30 min	1-10 copies/reaction	No	[13]
NATflow	RPA + Lateral flow assay (LFA), colorimetry	Integrated cartridge	16, 18	45 min	500-1000 copies/reaction	Yes	[14]
R-CHIP	Multiplex RPA + CRISPR-Cas12a, fluorescence	Hand-driven microfluidic chip	16, 18	~50 min	10^{-17} M (HPV16) 10^{-18} M (HPV18)	Yes (Smartphone)	[15]
**Ccard**	**Single-step RPA/CRISPR-Cas13a, fluorescence**	**Microfluidic chip + IoT device**	**16, 18, GAPDH**	**~30 min**	**5 copies/μL**	**Yes (Smartphone)**	**This work**

References

[1] Z.T.F. Yu, M.K. Cheung, S.X. Liu, J. Fu, Accelerated Biofluid Filling in Complex Microfluidic Networks by Vacuum-Pressure Accelerated Movement (V-PAM), Small 12 (2016) 4521–4530. https://doi.org/10.1002/smll.201601231.

[2] H.Q. Nguyen, H.K. Bui, V.M. Phan, T.S. Seo, An internet of things-based point-of-care device for direct reverse-transcription-loop mediated isothermal amplification to identify SARS-CoV-2, Biosensors and Bioelectronics 195 (2022) 113655. https://doi.org/10.1016/j.bios.2021.113655.

[3] T. Shu, X. Yin, Q. Xiong, C. Hua, J. Bu, K. Yang, J. Zhao, Y. Liu, L. Zhu, C. Zhu, Lift-CM: An integrated lift-heater centrifugal microfluidic platform for point-of-care pathogen nucleic acid detection using isothermal amplification and CRISPR/Cas12a, Biosensors and Bioelectronics 274 (2025) 117178. https://doi.org/10.1016/j.bios.2025.117178.

[4] J. Fang, Q. Chen, M. Ran, R. Chen, W. Chen, J. Cui, J. Wang, K. Zhong, L. Shi, C. Lu, H. Jiang, RPA-CRISPR/Cas12a-coupled microfluidic biosensor enabling on-site, sensitive quantification of Vibrio parahaemolyticus, Biosensors and Bioelectronics 296 (2026) 118327. https://doi.org/10.1016/j.bios.2025.118327.

[5] H. Ge, J. Feng, L. Huang, Z. Luo, H. Ling, L. Ma, M. Wang, H. Chen, L. Ren, Development of a highly sensitive, high-throughput and automated CRISPR-based device for the contamination-free pathogen detection, Biosensors and Bioelectronics (2025) 117323. https://doi.org/10.1016/j.bios.2025.117323.

[6] A. Sen, M. Masetty, S. Weerakoon, C. Morris, J.S. Yadav, S. Apewokin, J. Trannguyen, M. Broom, A. Priye, Paper-based loop-mediated isothermal amplification and CRISPR integrated platform for on-site nucleic acid testing of pathogens, Biosensors and Bioelectronics 257 (2024) 116292. https://doi.org/10.1016/j.bios.2024.116292.

[7] J.S. Gootenberg, O.O. Abudayyeh, J.W. Lee, P. Essletzbichler, A.J. Dy, J. Joung, V. Verdine, N. Donghia, N.M. Daringer, C.A. Freije, C. Myhrvold, R.P. Bhattacharyya, J. Livny, A. Regev, E.V. Koonin, D.T. Hung, P.C. Sabeti, J.J. Collins, F. Zhang, Nucleic acid detection with CRISPR-Cas13a/C2c2, Science 356 (2017) 438–442. https://doi.org/10.1126/science.aam9321.

[8] J.S. Chen, E. Ma, L.B. Harrington, M. Da Costa, X. Tian, J.M. Palefsky, J.A. Doudna, CRISPR-Cas12a target binding unleashes indiscriminate single-stranded DNase activity, Science 360 (2018) 436–439. https://doi.org/10.1126/science.aar6245.

[9] K. Zhang, Q. Li, K. Wang, Q. Zhang, C. Ma, G. Yang, Y. Xie, M.G. Mauk, S. Fu, L. Chen, RPA-CRISPR-Cas-Mediated Dual Lateral Flow Assay for the Point-of-Care Testing of HPV16 and HPV18, Bioconjugate Chem. 35 (2024) 1797–1804. https://doi.org/10.1021/acs.bioconjchem.4c00375.

[10] A. Ghouneimy, Z. Ali, R. Aman, W. Jiang, M. Aouida, M. Mahfouz, CRISPR-Based Multiplex Detection of Human Papillomaviruses for One-Pot Point-of-Care

Diagnostics, ACS Synth. Biol. 13 (2024) 837–850. https://doi.org/10.1021/acssynbio.3c00655.

[11] F. Zhao, Y. Hu, Z. Fan, B. Huang, L. Wei, Y. Xie, Y. Huang, S. Mei, L. Wang, L. Wang, B. Ai, J. Fang, C. Liang, F. Xu, W. Tan, F. Guo, Rapid and sensitive one-tube detection of mpox virus using RPA-coupled CRISPR-Cas12 assay, Cell Reports Methods 3 (2023) 100620. https://doi.org/10.1016/j.crmeth.2023.100620.

[12] X. Zheng, Y. Li, M. Yuan, Y. Shen, S. Chen, G. Duan, Rapid detection of HPV16/18 based on a CRISPR-Cas13a/Cas12a dual-channel system, Anal. Methods 14 (2022) 5065–5075. https://doi.org/10.1039/D2AY01536F.

[13] S. Seely, J.-M. Zingg, P. Joshi, B. Slomovitz, M. Schlumbrecht, E. Kobetz, S. Deo, S. Daunert, Point-of-Care Molecular Test for the Detection of 14 High-Risk Genotypes of Human Papillomavirus in a Single Tube, Anal. Chem. 95 (2023) 13488–13496. https://doi.org/10.1021/acs.analchem.3c01723.

[14] K.A. Kundrod, M. Barra, A. Wilkinson, C.A. Smith, M.E. Natoli, M.M. Chang, J.B. Coole, A. Santhanaraj, C. Lorenzoni, C. Mavume, H. Atif, J.R. Montealegre, M.E. Scheurer, P.E. Castle, K.M. Schmeler, R.R. Richards-Kortum, An integrated isothermal nucleic acid amplification test to detect HPV16 and HPV18 DNA in resource-limited settings, SCIENCE TRANSLATIONAL MEDICINE (n.d.).

[15] T. Xu, Y. Zhang, S. Li, C. Dai, H. Wei, D. Chen, Y. Zhao, H. Liu, D. Li, P. Chen, B. Liu, Y. Tian, Deep Learning-Enhanced Hand-Driven Microfluidic Chip for Multiplexed Nucleic Acid Detection Based on RPA/CRISPR, Advanced Science 12 (2025) 2414918. https://doi.org/10.1002/advs.202414918.
